# Banana MabHLH28 positively regulates the expression of softening-related genes to mediate fruit ripening independently or via cooperating with MaWRKY49/111

**DOI:** 10.1093/hr/uhae053

**Published:** 2024-02-23

**Authors:** Chaojie Wu, Danling Cai, Jun Li, Zengxiang Lin, Wei Wei, Wei Shan, Jianye Chen, Wangjin Lu, Xinguo Su, Jianfei Kuang

**Affiliations:** State Key Laboratory for Conservation and Utilization of Subtropical Agro-bioresources/Guangdong Provincial Key Laboratory of Postharvest Science of Fruits and Vegetables/Engineering Research Center of Southern Horticultural Products Preservation, Ministry of Education, College of Horticulture, South China Agricultural University, Guangzhou 510642, China; State Key Laboratory for Conservation and Utilization of Subtropical Agro-bioresources/Guangdong Provincial Key Laboratory of Postharvest Science of Fruits and Vegetables/Engineering Research Center of Southern Horticultural Products Preservation, Ministry of Education, College of Horticulture, South China Agricultural University, Guangzhou 510642, China; State Key Laboratory for Conservation and Utilization of Subtropical Agro-bioresources/Guangdong Provincial Key Laboratory of Postharvest Science of Fruits and Vegetables/Engineering Research Center of Southern Horticultural Products Preservation, Ministry of Education, College of Horticulture, South China Agricultural University, Guangzhou 510642, China; State Key Laboratory for Conservation and Utilization of Subtropical Agro-bioresources/Guangdong Provincial Key Laboratory of Postharvest Science of Fruits and Vegetables/Engineering Research Center of Southern Horticultural Products Preservation, Ministry of Education, College of Horticulture, South China Agricultural University, Guangzhou 510642, China; State Key Laboratory for Conservation and Utilization of Subtropical Agro-bioresources/Guangdong Provincial Key Laboratory of Postharvest Science of Fruits and Vegetables/Engineering Research Center of Southern Horticultural Products Preservation, Ministry of Education, College of Horticulture, South China Agricultural University, Guangzhou 510642, China; State Key Laboratory for Conservation and Utilization of Subtropical Agro-bioresources/Guangdong Provincial Key Laboratory of Postharvest Science of Fruits and Vegetables/Engineering Research Center of Southern Horticultural Products Preservation, Ministry of Education, College of Horticulture, South China Agricultural University, Guangzhou 510642, China; State Key Laboratory for Conservation and Utilization of Subtropical Agro-bioresources/Guangdong Provincial Key Laboratory of Postharvest Science of Fruits and Vegetables/Engineering Research Center of Southern Horticultural Products Preservation, Ministry of Education, College of Horticulture, South China Agricultural University, Guangzhou 510642, China; State Key Laboratory for Conservation and Utilization of Subtropical Agro-bioresources/Guangdong Provincial Key Laboratory of Postharvest Science of Fruits and Vegetables/Engineering Research Center of Southern Horticultural Products Preservation, Ministry of Education, College of Horticulture, South China Agricultural University, Guangzhou 510642, China; Agronomy Dean, Guangdong AIB Polytechnic College, Guangzhou 510507, China; State Key Laboratory for Conservation and Utilization of Subtropical Agro-bioresources/Guangdong Provincial Key Laboratory of Postharvest Science of Fruits and Vegetables/Engineering Research Center of Southern Horticultural Products Preservation, Ministry of Education, College of Horticulture, South China Agricultural University, Guangzhou 510642, China

## Abstract

Texture softening is a physiological indicator of fruit ripening, which eventually contributes to fruit quality and the consumer’s acceptance. Despite great progress having been made in identification of the genes related to fruit softening, the upstream transcriptional regulatory pathways of these softening-related genes are not fully elucidated. Here, a novel *bHLH* gene, designated as *MabHLH28*, was identified because of its significant upregulation in banana fruit ripening. DAP-Seq analysis revealed that MabHLH28 bound to the core sequence of ‘CAYGTG’ presented in promoter regions of fruit softening-associated genes, such as the genes related to cell wall modification (*MaPG3*, *MaPE1*, *MaPL5*, *MaPL8*, *MaEXP1*, *MaEXP2*, *MaEXPA2*, and *MaEXPA15*) and starch degradation (*MaGWD1* and *MaLSF2*), and these bindings were validated by EMSA and DLR assays. Transient overexpression and knockdown of *MabHLH28* in banana fruit resulted in up- and down-regulation of softening-related genes, thereby hastening and postponing fruit ripening. Furthermore, overexpression of *MabHLH28* in tomato accelerated the ripening process by elevating the accumulation of softening-associated genes. In addition, MabHLH28 showed interaction withMaWRKY49/111 and itself to form protein complexes, which could combinatorically strengthen the transcription of softening-associated genes. Taken together, our findings suggest that MabHLH28 mediates fruit softening by upregulating the expression of softening-related genes either alone or in combination with MaWRKY49/111.

## Introduction

Fruit softening that takes place in fruit ripening and postharvest preservation is a critical ripening indicator determining fruit quality, storage life, economic value in the market, as well as the consumer’s acceptance and satisfaction [1]. However, over-softening has a negative effect on fruit storability, which makes the fruit sensitive to mechanical damage and microorganism infection, thus bringing about problems in postharvest handling during transportation and marketing [2]. In general, fruit softening is as a result of sequential disassembling and breakdown of cell wall polysaccharides, which is cooperatively regulated by cell wall hydrolytic enzymes, such as polygalacturonase (PG), pectin methyl esterase (PME), pectinesterase (PE), pectate lyase (PL), endo-1,4-beta-glucanase (EGase), β-galactosidase (β-GAL), xyloglucan endo-transglucosylase/hydrolase (XTH), mannanase (MAN), and Expansin [3]. Additionally, in some fruits such as banana and kiwifruit, texture softening is also provoked because of the degradation of starch molecules that are produced in the process of fruit development [4, 5]. Therefore, regulation of fruit softening is the major theme of intensive investigation for improving comprehensive benefits of fruit crops by farmers, engineers, and scientists.

Fruit softening is modulated by both environmental stimuli and intrinsic developmental cues, which lead to a variety of signalling pathways ultimately resulting in transcriptional responses. Transcription factors (TFs) are the central nodes in various signalling cascades, which integrate various external and internal signals to optimize downstream transcriptional reprogramming by transcriptional modulation of a large number of specific target genes [6]. The precise transcriptional mechanisms rely on how closely related TFs modulate specific biological events, which is determined by primary DNA sequence and structural characteristics of TFs, and how heteromeric complex formations influence functional specificity, which results from the ability of TFs to form higher-order protein complexes [7]. In fruits, many TFs, such as MCM1, Agamous, Deficiens and SRF (MADS), Ethylene Response Factor (ERF), basic Leucine Zipper (bZIP), Brassinazole Resistant (BZR), Lateral Organ Boundaries (LOB), Ethylene Insensitive 3-Like (EIL), and basic-Helix–Loop–Helix (bHLH), have been identified to mediate transcriptional cascades leading to fruit softening [8–12]. The bHLH proteins represent a large TF family in all eukaryotes including plants, which are defined by a common bHLH structure consisting of 50 to 60 amino acids [13]. A standard bHLH domain is usually composed of two parts, including a basic region positioning at the N-terminus of bHLH domain, which is known to recognize and bind to the E-box consensus (CANNTG), followed by a HLH region positioning at the C-terminus of the bHLH domain that is accountable for homo- and heterodimer formation [14]. The bHLH TFs engage in a multitude of physiological events, from plant growth and development (e.g., embryo growth, anthers development, fruit dehiscence, and seed dispersal) to stress adaptation (e.g., pathogen infection, cold stress, and salt stress) [15]. Currently, some bHLH proteins that are involved in fruit ripening regulation have been characterized. For example, overexpression of *SlbHLH22* in tomato reduced the shelf life of the transgenic fruit, possibly through up-regulation of the cell wall metabolic genes consisting of *SlMAN*, *lipoxygenase A* (*SlLoxA*), *SlEXP1*, *SlXTH5*, and *SlPE* [16]. Another bHLH TF in tomato, SlbHLH95, which acts as one of the downstream targets of RIN, modulates fruit ripening via influencing expression of ripening-associated genes [17]. Apple fruit expressing *MdbHLH3* promotes fruit softening by elevating the degradation of cell wall components, suggesting that MabHLH3 negatively affects postharvest storage [18]. The process by which CpbHLH1/2 regulate the transcription of downstream carotenogenic genes is mostly related to carotenoid formation during fruit ripening [19]. However, by comparing with the extensive research of bHLH TFs in model plants, the roles of different bHLHs in terms of fruit softening remain largely unknown.

WRKY TFs, named after their specific conserved heptapeptide sequence WRKYGOK, are a class of TFs unique to plants and widely involved in fruit ripening, leaf senescence, hormone signalling, and responses to biotic and abiotic stresses [20]. WRKY TFs are mainly involved in regulating the expression of target genes by specifically binding to *cis*-acting elements (T)TGAC(C/T) (W-box) of their promoters to activate or repress their transcriptional activities, thus regulating the processes of plant growth and development [21]. In kiwifruit fruit, AcWRKY40 binds to the promoters of genes encoding S-adenosyl-L-methionine (AcSAM2), 1-amino-cyclopropane-1-carboxylic acid synthase 1 (AcACS1), and AcACS2 to activate them and participate in postharvest ripening of kiwifruit [22]. In strawberry fruit, FvWRKY48 binds to the *FvPLA* promoter to control fruit softening [23]. In apple fruit, MdWRKY126 regulates malate accumulation by activating the expression of *cytosolic malate dehydrogenase 5* (*MdMDH5*) [24]. In banana fruit, MaWRKY49/111 bind to and activate the *1-amino-cyclopropane-1-carboxylic acid oxidase 1* (*MaACO1*) and *MaACS1* promoters [25], although studies have shown that WRKY TFs play an important role in fruit ripening and softening. However, the current understanding of WRKY-interacting ripening-related TFs is limited, and their specific regulatory mechanisms are unknown.

Protein–protein interaction is crucial in cellular communication in diverse biological events, and characterization of the interaction networks could yield useful information on understanding the functions of given proteins [26]. It has been reported that TFs act in multimeric complexes consisting of members of different TF families and other proteins, which substantially influences their binding specificity and the transcriptional activity of their target genes. For example, citrus NAM-ATAF1/2-CUC2 62 (CitNAC62) associates with CitWRKY1 to additively stimulate the expression of *aconitase 3* (*CitAco3*), a citrate degradation-related gene [27]. Litchi LcR1MYB1 reserves the transcriptional effect of LcNAC13 on the transcription of anthocyanin formation genes through protein–protein interaction [28]. In papaya fruit, Auxin Response Factor 2 (CpARF2) interacts with CpEIL1 to enhance CpEIL1-mediated transcription of ripening-associated genes [29]. Interaction between indole-3-acetic acid 1 (PpIAA1) and PpERF4 mediates peach fruit ripening by upregulating the ripening-related gene expression [30]. More recently, MaNAC083 showed interaction with MaMADS1 to regulate ethylene production by modulating the transcription of *MaACS1* and *MaACO1/4/5/8* [31]. Moreover, studies also showed that bHLH TFs could form protein complexes with themselves or other transcriptional regulators to impact the transcriptional activity and target specificity of bHLH proteins [32, 33]. For instance, in kiwifruit, the combination of AcMYB123 and AcbHLH42 has a substantial effect on activation of anthocyanin biosynthetic genes such as *anthocyanidin synthase* (*AcANS*) and *UDP-3-O-galactosyltransferase 1* (*AcF3GT1*) [34]. Similarly, citrus CitbHLH2 interacts with CitMYB52 to synergistically enhance *aluminum-activated malate transporter* (*CitALMT*) transcription, thus negatively modulating citrate accumulation [35]. In the case of strawberry, FvbHLH9 interacts with Elongated Hypocotyl 5 (FvHY5) to further enhance the transcription of *dihydroflavonol 4-reductase gene* (*FvDFR*), a key enzyme gene in anthocyanin production [36]. Given that protein–protein interactions have an obvious impact on TF activity, it is very important to unravel the possible interaction partners of bHLH proteins, which allows us to fully understand the functional mechanisms of bHLHs.

**Figure 1:**
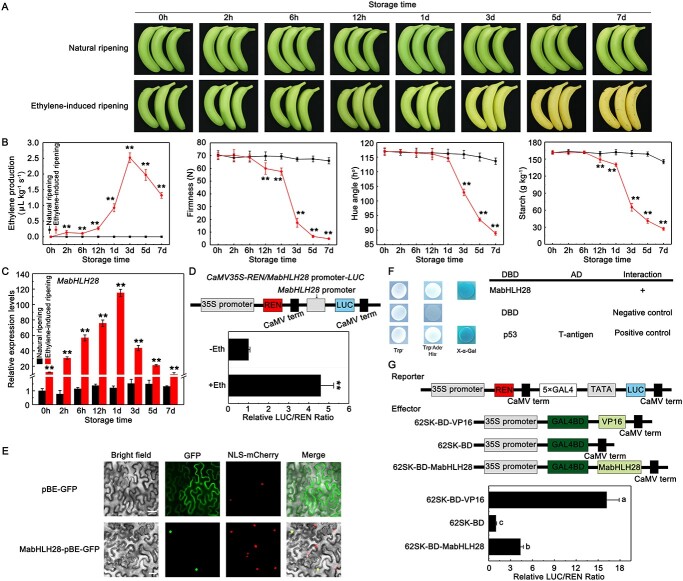
Physiological changes of banana fruit and molecular characterization of MabHLH28. (**A**) Phenotype of bananas under natural ripening and ethylene-induced ripening. (**B**) Variation of ethylene production, fruit firmness, hue angle, and starch content during ripening. Data represent the mean ± SE of six biological replicates (^**^P < 0.01). (**C**) Expression of *MabHLH28* in banana fruit with and without (control) ethylene treatment. Relative expression level of *MabHLH28* was analysed RT-qPCR. Each value is mean ± SE of three replicates (^**^P < 0.01). (**D**) *MabHLH28* promoter activity in response to ethylene. Each value is mean ± SE of six replicates (^**^P < 0.01). (**E**) Subcellular localization of MabHLH28 in tobacco leaf cells. NLS-mCherry serves as a nuclear localization signal marker; bar, 25 μm. (**F**) Transcriptional activity of MabHLH28 in yeast cells. The pGBKT7 empty vector (BD) was used as a negative control and pGBKT7–53 + pGADT7-T as a positive control. (**G**) Transactivation of MabHLH28 in tobacco leaf cells. The ratio of LUC/REN of the empty 62SK-BD vector (negative control) was set to 1. 62SK-BD-VP16 was used as a positive control. Each value is mean ± SE of six replicates. Different letters indicate significant differences at the *P* < 0.05 level.

Banana is a popular fruit consumed throughout the world, due to its good sources of health-promoting compounds, such as bioactive phenols, potassium, carotenoids, and dietary fiber [37]. Banana fruit are commonly cut from half to fully developed stages, and afterwards transported to consumer countries where artificial ripening is facilitated by ethylene or ethylene-releasing compounds under the controlled circumstances. Proper temperature, humidity, maturity, ethylene gas, and treatment time are all necessary for the ripening of banana fruit. During ripening, the quality traits develop, which include firmness loss, aroma production, sugar accumulation, and colour change. However, the ripening-caused short shelf-life of banana fruit is a persistent challenge worldwide, especially in regions where the postharvest handling and storage infrastructure are insufficient. Numerous postharvest practices, such as refrigerated storage, modified atmosphere storage, edible coating, and application of chemicals like GA, NO, and H_2_S, are reported to keep up the quality of banana fruit, but these technologies are not widely applied in the banana industry because of complicated skill, high cost, or food safety concerns. To this end, it would be interesting to investigate the transcriptional regulatory hierarchy related to banana fruit ripening and softening, which has the potential to help improve postharvest quality and shelf-life of the horticultural crops in practice. Our previous works have revealed that banana MabHLH6 is associated with fruit softening by promoting the expression of a handful of starch degrading genes [4], while MabHLH7 mediates fruit softening through stimulating the transcription of a group of cell wall modifying genes [38]. These findings suggest that different bHLHs may have different effects on fruit softening by targeting specific genes. As there are at least 259 *bHLH* genes in the genome of *Musa acuminate* [39], the involvement of other members of bHLH TFs in fruit softening regulation is yet to be defined.

In this study, a novel up-regulated *bHLH* TF in fruit ripening named *MabHLH28* was isolated from the previously published transcriptomic data related to banana fruit ripening [40]. Then, the binding motif bound by MabHLH28 was characterized, and MabHLH28’s target genes were studied at a genome scale. Moreover, the biological functions of *MabHLH28* in fruit ripening were investigated by transient overexpression and virus-induced gene silencing (VIGS) of *MabHLH28* in banana fruit as well as stable overexpression in tomato fruit. Additionally, the possible interaction partners of MabHLH28 were also identified, in order to fully understand the complex network of MabHLH28 in banana fruit softening. We bring evidence that MabHLH28 is a positive regulator of fruit softening by promoting the transcription of cell wall modification and starch degradation-related genes independently or via interaction with MaWRKY49/111, which provide a foundation for building the transcriptional pathways in banana fruit softening.

**Figure 2:**
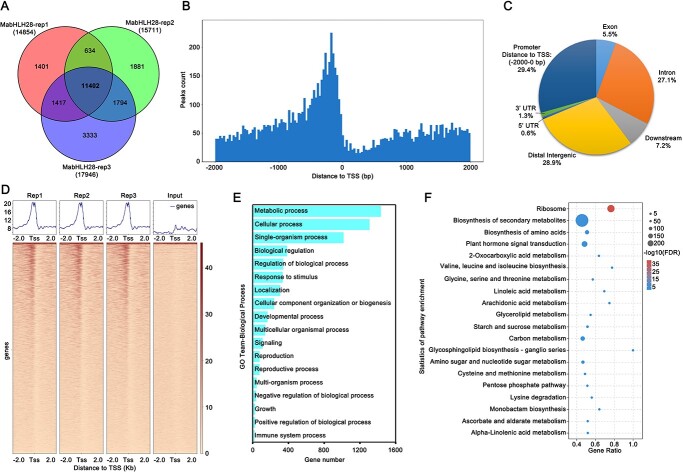
Genome-wide study of MabHLH28-binding sites by DAP-Seq. (**A**) DAP-Seq using three biological replicates revealed 11 042 highly-confident MabHLH28 binding peaks. (**B**) MabHLH28-binding sites are highly enriched in proximal to the transcriptional start sites. (**C**) Distribution of the enriched MabHLH28-binding sites within genic regions, including promoter (−2000 bp), downstream, 5$ ^{\prime} $ UTR, 3$ ^{\prime} $ UTR, Exon, Intron, and Distal Intergenic regions. (**D**) Mean density map and heat map assay of MabHLH28 binding genes. (**E**, **F**) GO biological process (**E**) and KEGG pathway enrichment analysis (**F**) of MabHLH28-binding genes.

## Results

### Identification and characterization of MabHLH28 from banana fruit

Given the significance of TFs in fruit ripening, characterization of bHLHs that specifically modulate fruit softening is important for a further understanding of fruit softening in banana. Based on our published RNA-Seq data related to banana fruit ripening [40], a *bHLH* gene, which was designated as *MabHLH28* in a previous report [39], was identified and isolated because of its significant upregulation in the ripening. The entire cDNA of *MabHLH28* (Gene ID: Ma02_g11480) was cloned and sequenced. A phylogenetic analysis indicated that MabHLH28 displayed a closer relationship with Arabidopsis AtbHLH049 and AtbHLH063 proteins, belonging to the GBOF subgroup (Fig. S1A, see online supplementary material). Additionally, MabHLH28 shares a conserved bHLH structure with other bHLH proteins from Arabidopsis (Fig. S1B, see online supplementary material), which is a typical feature of bHLH proteins. To further characterize the involvement of *MabHLH28* in fruit ripening, mature-green banana fruit were treated with exogenous ethylene. Compared with the untreated group, ethylene application hastened the ripening of banana fruit (Fig. 1A). The maximal ethylene yield in the ethylene-treated fruit appeared at 3 days after treatment, with fruit firmness, hue angle, and starch content declining faster compared to the untreated control (Fig. 1B). Meanwhile, we used RT-qPCR assays to monitor the expression pattern of *MabHLH28* in banana fruit with and without (control) ethylene treatment. In line with the findings of the transcriptome data [32], *MabHLH28* transcript increased significantly in banana fruit treated with ethylene, and no significant change was observed in the control fruit (Fig. 1C). Furthermore, *MabHLH28* also displayed the maximal levels in the ripening stage of banana fruit under natural, ethylene-induced, and 1-MCP-delayed ripening, respectively (Fig. S2, see online supplementary material). In addition, promoter activity assay showed that ethylene stimulated the promoter activity of *MabHLH28* (Fig. 1D), implying that *MabHLH28* is ethylene-responsive. Fluorescence of MabHLH28-GFP in tobacco leaf cells exclusively targeted to the nucleus, which co-localized with that of the NLS-mCherry (Fig. 1E), suggesting that MabHLH28 fits with the TF attribute. To determine if MabHLH28 has transactivation activity, we firstly performed yeast system. As illustrated in Fig. 1F, yeast cells transforming pGBKT7-MabHLH28 but not the pGBKT7 alone (negative control) were able to grow in the selective dropout medium SD/−Trp/−Ade/-His, and show blue colour at the presence of X-α-Gal, suggesting that MabHLH28 functions as a transcriptional activator in yeast cells. Then, a GAL4-dependent chimeric transactivation assay in tobacco epidermal cells was performed to validate the transcriptional activity of MabHLH28. As shown in Fig. 1G, MabHLH28 could activate the reporter expression *in vivo*, which is consistent with the findings of yeast system (Fig. 1F). These observations suggest that the ripening-induced MabHLH28 is a nucleus-localized activator that could activate the expression of the downstream target genes.

### Identification of MabHLH28’s target genes at genome-wide scale

To gain genome-wide insight into the MabHLH28’s regulatory network, we applied a DNA-affinity purification sequencing (DAP-Seq) approach, which is an *in vitro* TF-binding discovery assay [41]. Analysis of MabHLH28 DAP-Seq data with three biological replicates revealed a total of 11 402 reproducible binding sites (Fig. 2A), which are regarded as high-probability MabHLH28 target genes for the following analysis. As expected for a TF, MabHLH28-binding sites are highly located in the regions upstream of the transcription start site (TSS) (Fig. 2B), highlighting the roles of MabHLH28 in gene expression regulation. Particularly, MabHLH28-binding peaks are distributed in promoter regions (29.4%), intergenic regions (28.9%), intron regions (27.1%), downstream regions (7.2%), exon regions (5.5%), 3′-untranslated regions (UTR, 1.3%), and 5′-UTR regions (0.6%) (Fig. 2C). Based on the findings of mean density map and heat map assay, the control input shows a very weak binding signal and tends to be horizontal, while the MabHLH28 binding signal is the strongest in the area adjacent to TSS, indicating that the quality of DAP-Seq experiments is highly faithful (Fig. 2D). GO and KEGG classification analysis showed that MabHLH28’s putative target genes took part in a variety of biological processes, particularly in the regulation of metabolic process (Fig. 2E and F), implying that MabHLH28 might regulate the metabolic processes that affect banana fruit ripening.

### MabHLH28 targets the cell wall modifying and starch degrading genes

**Figure 3:**
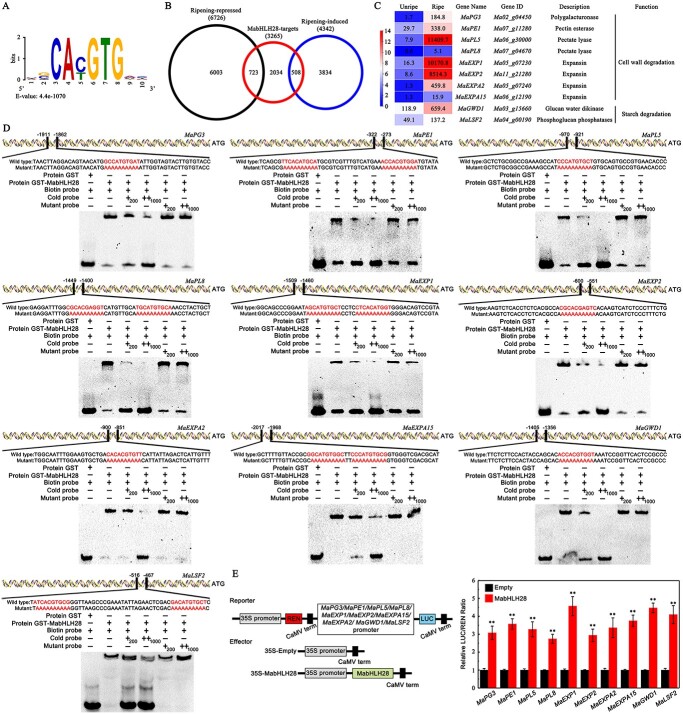
Characterization of MabHLH28-binding ripening-associated genes. (**A**) The potential motif enriched in MabHLH28 target genes. (**B**) Venn diagram showing the overlap of genes bound by MabHLH28 and the genes differentially expressed during banana fruit ripening based on RNA-seq. (**C**) Differentially expressed MabHLH28-targeted softening-related genes in unripe and ripe banana fruit. (**D**) EMSA assays showed that MabHLH28 was associated with the promoters of softening-related genes (*MaPG3*, *MaPE1*, *MaPL5*, *MaPL8*, *MaEXP1*, *MaEXP2*, *MaEXPA2*, *MaEXPA15*, *MaGWD1*, and *MaLSF2*). Two hundred- and 1000-fold concentrations of the unlabeled cold or mutant probes were used for the competition test. (**E**) DLR assay showing that MabHLH28 activates the transcription of softening related genes. The ratio of LUC/REN of the empty 62-SK vector (negative control) was set to 1. Each value represents mean ± SE of six biological replicates (^**^P < 0.01).

To determine the possible binding sites of MabHLH28, MEME-ChIP analysis [42] was performed. As expected, we identified an identical motif for MabHLH28, with core sequence of CA(C/T)GTG (Fig. 3A), which strongly resembles with G-box (CACGTG) and E-box (CANNTG). Analysis of the positions of this binding motif together with the RNA-Seq revealed that several genes encoding cell wall hydrolytic enzymes such as *MaPG3*, *MaPE1*, *MaPL5*, *MaPL8*, *MaEXP1*, *MaEXP2*, *MaEXPA2*, and *MaEXPA15*, as well as the genes involved in starch breakdown such as *α-glucan water dikinase 1* (*MaGWD1*) and *Like SEX4 isoform 2* (*MaLSF2*) contained one or more MabHLH28 binding motifs in their promoters (Fig. 3B and C), implying that MabHLH28 modulates banana fruit ripening via targeting these fruit softening-related genes. To test if MabHLH28 directly binds to these promoter regions, we conducted electrophoretic mobility shift assays (EMSAs). The probes from the *MaPG3*, *MaPE1*, *MaPL5*, *MaPL8*, *MaEXP1*, *MaEXP2*, *MaEXPA2*, *MaEXPA15*, *MaGWD1*, and *MaLSF2* promoters were labelled with biotin and incubated with the MabHLH28 protein produced in *Escherichia coli* (Fig. S3, see online supplementary material). As seen in Fig. 3D, the GST-MabHLH28 protein was able to recognize the regions of these promoters covering the MabHLH28-binding motifs, but it could not bind efficiently to these probes where the MabHLH28-binding motifs were mutated (Fig. S4, see online supplementary material). The observed binding was gradually decreased in a dose-dependent manner with the addition of the increasing concentrations of unlabelled same sequences but not mutated sequences, suggesting the specificity of binding. To assess whether MabHLH28 imposes the transcriptional activation of these softening-associated genes, we conducted a transient transactivation assay. Co-expression of *MabHLH28* with *LUC* driven by any promoter of the softening-associated genes tested in tobacco leaves resulted in elevated levels of LUC activity (Fig. 3E), suggesting that MabHLH28 imposes an activated effect on the transcription of these genes. Overall, these results indicate that MabHLH28 promotes transcription of softening-associated genes by direct promoter binding.

### Transient overexpression and knockdown of *MabHLH28* expression affect banana fruit ripening and softening

**Figure 4:**
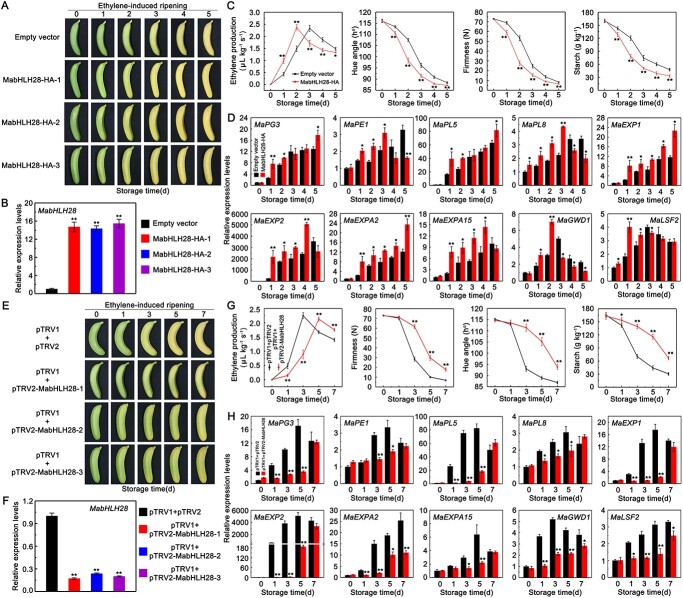
Functional analysis of *MabHLH28* in banana fruit. (**A**) Phenotype of banana fruit transiently overexpressing *MabHLH28* and empty vector during the ethylene-induced ripening. (**B**) qRT-PCR analysis showed the expression of *MabHLH28* in *MabHLH28*-overexpression and control banana fruit at 0 d in (**A**). (**C**) Changes of ethylene production, fruit firmness, colour index, and starch content in *MabHLH28*-overexpression and control fruit. Each value represents the mean ± SE of six replicates (^**^P < 0.01 and ^*^P < 0.05). (**D**) Relative expression of *MaPG3*, *MaPE1*, *MaPL5*, *MaPL8*, *MaEXP1*, *MaEXP2*, *MaEXPA2*, *MaEXPA15*, *MaGWD1*, and *MaLSF2* in *MabHLH28*-overexpression and control banana fruit. Each value represents the mean ± SE of three biological replicates (^**^P < 0.01 and ^*^P < 0.05). (**E**) Phenotype of banana fruit transiently silencing *MabHLH28* and control fruit during the ethylene-induced ripening. (**F**) qRT-PCR analysis showed the expression of *MabHLH28* in *MabHLH28*-silencing and control banana fruit at Day 0 in (**E**). (**G**) Changes of ethylene production, fruit firmness, colour index, and starch content in *MabHLH28*-silencing and control fruit. Each value represents the mean ± SE of six replicates (^**^P < 0.01 and ^*^P < 0.05). (**H**) Relative expression of *MaPG3*, *MaPE1*, *MaPL5*, *MaPL8*, *MaEXP1*, *MaEXP2*, *MaEXPA2*, *MaEXPA15*, *MaGWD1*, and *MaLSF2* in *MabHLH28*-silencing and control banana fruit. Each value represents the mean ± SE of three biological replicates (^**^P < 0.01 and ^*^P < 0.05).

Due to the difficulties in stable transformation of banana fruit, we investigated the functions of MabHLH28 by transient overexpression and virus-induced gene silencing (VIGS) of *MabHLH28* in banana fruit, respectively. First, we transiently expressed *35S::MabHLH28-HA* in unripe banana fruit, and then the infiltrated and control fruit were ripened by ethylene (Fig. 4A). The RT-qPCR assay indicated the high abundance of *MabHLH28* in transgenic fruit but not in the control (Fig. 4B), suggesting the success of overexpression. We found that transient overexpression of *MabHLH28* could hasten fruit ripening and softening, as checked by earlier ethylene maximal production, and faster drop of fruit firmness, hue angle, and starch content in *MabHLH28*-overexpression fruit than in the control fruit (Fig. 4C). Moreover, RT-qPCR analyses indicated that softening-related genes such as *MaPG3*, *MaPE1*, *MaPL5*, *MaPL8*, *MaEXP1*, *MaEXP2*, *MaEXPA2*, *MaEXPA15*, *MaGWD1*, and *MaLSF2* were substantially up-regulated in banana fruit expressing *MabHLH28* (Fig. 4D), which is consistent with the phenotypes.

To further validate the function of *MabHLH28* in banana fruit softening, we used VIGS approach to transiently inhibit *MabHLH28* expression in banana fruit (Fig. 4E and F). As seen in Fig. 4G, silencing of *MabHLH28* delayed fruit ripening and softening, showing later occurrence of ethylene peak, and higher fruit firmness, hue angle and starch content. Expression of *MaPG3*, *MaPE1*, *MaPL5*, *MaPL8*, *MaEXP1*, *MaEXP2*, *MaEXPA2*, *MaEXPA15*, *MaGWD1*, and *MaLSF2* was significantly down-regulated in banana fruit infiltrated with TRV1 and TRV2-MabHLH28 compared to the control fruit transformed with the TRV1 and TRV2 vectors (Fig. 4H). Altogether, these observations demonstrate that MabHLH28 has a positive role in fruit ripening and softening via upregulating expression of softening-associated genes.

### Overexpression of *MabHLH28* in tomato accelerates fruit ripening and softening

**Figure 5:**
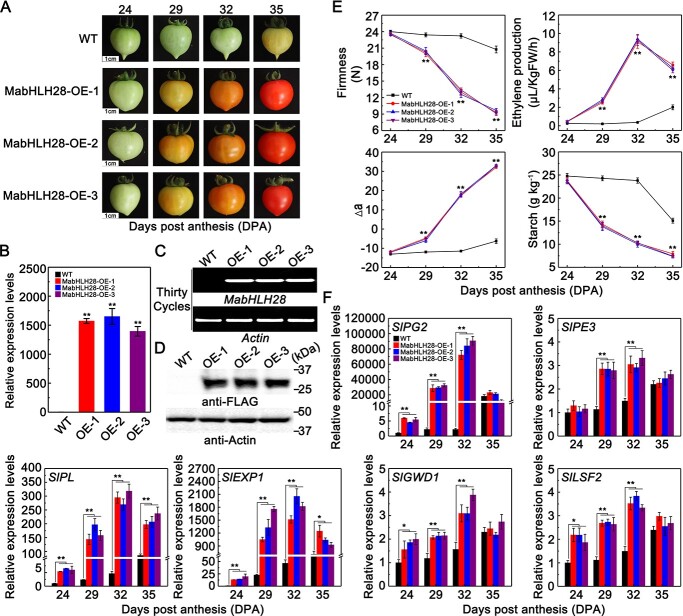
Overexpression of *MabHLH28* in tomato promoted fruit ripening. (**A**) Fruit ripening process of wild-type (WT) and *OE-MabHLH28* lines. Fruit at 24, 29, 32, and 35 days post-anthesis (DPA) from WT and three independent *OE-MabHLH28* lines (*OE-1*, *OE-2*, and *OE-3*) are shown. (**B**–**D**) The expression of *MabHLH28* in WT and *OE-MabHLH28* lines as determined by RT-qPCR (**B**), semi-RT-PCR (**C**), and western blot (**D**) analysis. Pericarp tissues of WT and OE-MabHLH28 lines at 29 DPA were used for detection. Thirty cycles of semi-RT-PCR were used in transgenic tomato identification. For western blot analysis, total proteins were extracted and detected by anti-FLAG antibody. *Actin* was used as an internal reference. (**E**) Changes in ethylene production, fruit firmness, peel chroma, and starch content in WT and *OE-MabHLH28* lines during ripening. Each value represents the means ± SE of six replicates. (**F**) Relative expression of *SlPG2*, *SlPL*, *SlPE3*, *SlEXP1*, *SlGWD1*, and *SlLSF2* in WT and *OE-MabHLH28* fruit during ripening. The transcript levels were determined by qRT-PCR using *SlActin* as the reference gene, and each gene was expressed as a ratio relative to the WT at 24 DPA. Each value denotes mean ± SE of three biological replicates (^**^P < 0.01 and ^*^P < 0.05).

In order to investigate the contribution of MabHLH28 to fruit ripening, we carried out a further genetic analysis by overexpression of *MabHLH28* in ‘Micro-Tom’ tomato fruit (Fig. 5A). The mRNA and protein levels of MabHLH28 in wild-type (WT) and OE-MabHLH28 lines of tomato were detected by RT-qPCR, semi-RT-PCR, and western blot assays (Fig. 5B–D), and three independently transgenic lines (OE-1, OE-2, and OE-3) were chosen for subsequent study. Strikingly, the difference in ripening phenotypes between *OE-MabHLH28* and WT fruits was obvious at 29 days post anthesis (DPA). At that moment, fruit colour was broken in *OE-MabHLH28* tomatoes, whereas that of the WT fruit was still green. Moreover, the fruit of *OE-MabHLH28* reached red-ripening stage at 35 DPA, while WT fruit had just begun to change color. Meanwhile, compared with WT fruit, the firmness of *MabHLH28-*overexpression fruit began to decline rapidly at 29 DPA, and the ethylene production reached a peak at 32 DPA (Fig. 5E). Additionally, compared with WT fruit, expression of the softening-related genes in tomato fruit, such as *SlPG2*, *SlPL*, *SlPE3*, *SlEXP1*, *SlGWD1*, and *SlLSF2*, was significantly up-regulated in the transgenic fruit at 29 DPA (Fig. 5F). Taken together, these data indicate that MabHLH28 positively modulates fruit ripening and softening by elevating the expression of softening-related genes.

### MabHLH28 interacts with MaWRKY49/111 to synergistically activate transcription of softening-associated genes

**Figure 6:**
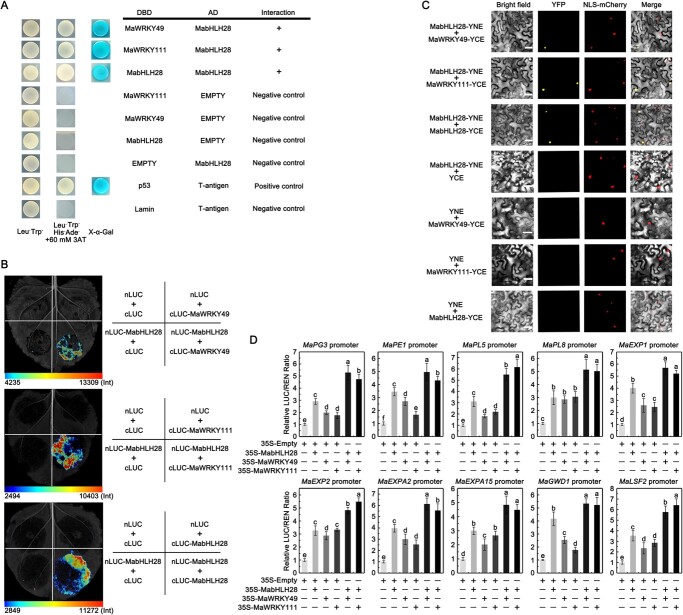
MabHLH28 physically interacts with MaWRKY49/111 and itself. (**A**) Y2H assay showing that MabHLH28 could interact with MaWRKY49/111 and MabHLH28. Different combinations were co-transformed into the yeast strain Gold Y2H. The ability of yeast cells to grow on selection media for possible interaction assessment. The T-antigen and p53 were used as the positive control. (**B**) LCI assay showing the interaction between MabHLH28 and MaWRKY49/111, MabHLH28. nLUC-MabHLH28 was co-expressed with cLUC-MaWRKY49/111 and cLUC-MabHLH28; MabHLH28-nLUC/cLUC, nLUC/cLUC-MaWRKY49/111, nLUC/cLUC-MabHLH28 and nLUC/cLUC were used as negative controls. The Luciferase activity was detected at 72 h post-infiltration. The calibration bar on the bottom indicates the representative images and quantification of luciferase activity in tobacco leaves.(**C**) BiFC assay showing the interaction between MabHLH28 and MaWRKY49/111, MabHLH28. MabHLH28 and MaWRKY49/111 were fused with the pUC-pSPYNE or the pUC-pSPYCE. Expression of MabHLH28 or MaWRKY49/111 alone with the empty vector was used as negative controls. NLS-mCherry was taken as a nuclear marker. Bar, 25 μm. (**D**) DLR assay showing that association between MabHLH28 and MaWRKY49/111 further elevated the transcription of *MaPG3*, *MaPE1*, *MaPL5*, *MaPL8*, *MaEXP1*, *MaEXP2*, *MaEXPA2*, *MaEXPA15*, *MaGWD1*, and *MaLSF2*. The ratio of LUC/REN of the empty 62-SK vector (negative control) was set to 1. Each value is the mean ± SE of six replicates. Different letters indicate significant differences at the *P* < 0.05 level.

The ethylene production was also affected in banana fruit expressing or silencing of *MabHLH28* (Fig. 4), suggesting the likelihood that MabHLH28 may directly or indirectly regulate ethylene biosynthesis by interacting with other TFs that target ethylene biosynthetic genes. Previously, MaWRKY49 and MaWRKY111 were reported to function in banana fruit ripening by targeting ethylene formation genes *MaACS1* and *MaACO1* [25]. To detect whether MabHLH28 interacts with MaWRKY49/111, we firstly performed yeast two-hybrid (Y2H) assays. It was found that MabHLH28 strongly interacted with MaWRKY49 and MaWRKY111 in Y2H assays (Fig. 6A). Moreover, we also found that MabHLH28 could interact with itself for homodimer formation, which is a typical nature of most bHLH TFs [13]. The luciferase complementation imaging (LCI) assay was used to prove the interaction between MabHLH28 and MaWRKY49/111 or MabHLH28 itself. As observed in Fig. 6B, LUC signal was observed when nLUC-MabHLH28 and cLUC-MaWRKY49, nLUC-MabHLH28 and cLUC-MaWRKY111, as well as nLUC-MabHLH28 and cLUC-MabHLH28 were co-transformed into tobacco leaf cells. Then, a bimolecular fluorescence complementation (BiFC) assay indicated that co-infiltration of *Nicotiana benthamiana* leaves with *Agrobacterium tumefaciens* strains expressing complementary MabHLH28-YFP (MabHLH28 auto-interaction), as well as complementary MabHLH28-YFP and MaWRKY49/111-YFP constructs (MabHLH28-MaWRKY49/111 interaction), elicited a strong YFP signal in the nuclei for each reciprocal combination (Fig. 6C). By contrast, the four negative controls failed to elicit YFP signal. These findings demonstrate the interaction between MabHLH28 and MaWRKY49/111 or itself.

The interaction between MabHLH28 and MaWRKY49/111 indicates that they may affect each other’s activity. To validate this possibility, we conducted a transient reporter gene assay. Data showed that MabHLH28 or MaWRKY49/11 alone had obvious activation on the target genes (2–4 folds higher than the control vector). However, when MabHLH28 and MaWRKY49/111 were co-expressed, we observed a significant activation effect on softening-associated gene transcription (more than 5-fold that of the control vector) (Fig. 6D). Given that MaWRKY49/111 act as activators of *MaACS1* and *MaACO1*, we also tested whether interaction between MabHLH28 and MaWRKY49/111 has an effect on *MaACS1* and *MaACO1* transcription. Similarly, a further increment of *MaACS1* and *MaACO1* transcription was observed when MabHLH28 and MaWRKY49/111 were co-expressed, comparing to the one only expression of single TF (Fig. S5, see online supplementary material). These findings support cooperative functions of MabHLH28 and MaWRKY49/111 in the regulation of softening-associated genes and ethylene biosynthetic genes.

## Discussion

During the process of fruit ripening, banana fruit undergo texture softening. It is well acknowledged that softening of banana fruit is primarily caused by decrease of peptic substances in cell wall and degradation of starch [43]. Previous reports indicated that expression of genes encoding cell wall hydrolysis proteins and starch breakdown enzymes is linked with fruit softening, but the transcriptional regulatory hierarchy of these genes remains largely unclear. To date, characterization of transcriptional regulators of these softening-related genes is a focused issue in postharvest biology, for the reason that manipulating the expression of distinct upstream regulator using genetic engineering strategy may have potential for prolonging the storage time of the fruit, as is in the case of SlLOB1 in tomato [44]. Numerous TFs have been identified in fruit softening regulation through targeting the promoters of softening-related genes in various fruits, such as CpERF9 in papaya [45], SlBES1 in tomato [46], DkBZR1/2 in kiwifruit [47], PpERF/ABR1 in peach [48], FvMYB79 in strawberry [49], and PavDof2/6/15 in sweet cherry [50].

The bHLH-type TFs are a large gene family in plants, with 162 members in Arabidopsis [51] and 259 members in banana [39]. Although some *bHLH* genes have been functionally identified in banana, such as MabHLH1-MabHLH7 [4, 38, 52], MabHLH060/183 [53], and MaMYC2a/b [54], the functions of the majority of banana bHLHs have not been uncovered. In this context, we identified a ripening-induced *bHLH* gene *MabHLH28* from the transcriptome related to banana ripening, which shows close phylogenetic relationship with Arabidopsis bHLH049 and bHLH063 that has a role in somatic embryogenesis [55]. We showed that MabHLH28 was targeted to nucleus and displayed transactivation capacity (Fig. 1) consistent with its role in being a TF. In agreement with this notion, DAP-Seq analysis revealed that MabHLH28 primarily recognized the promoter regions of the target genes (Fig. 2). Particularly, we characterized a putative binding motif for MabHLH28, with core sequence of CA(C/T)GTG (Fig. 3A), which resembles G-box (CACGTG) and E-box (CANNTG) [56]. Similarly, using the ChIP-Seq technology, the G-box (CACGTG) element was identified as PIF7 and bHLH60 binding sites [57]. Promoters of several softening-associated genes, such as *MaPG3*, *MaPE1*, *MaPL5*, *MaPL8*, *MaEXP1*, *MaEXP2*, *MaEXPA2*, *MaEXPA15*, *MaGWD1*, and *MaLSF2* harbour one or more MabHLH28-binding motifs, implying that MabHLH28 may target these genes to take part in fruit softening regulation. Consequently, EMSA and DLR analyses illustrated that MabHLH28 binds directly to the promoters of *MaPG3*, *MaPE1*, *MaPL5*, *MaPL8*, *MaEXP1*, *MaEXP2*, *MaEXPA2*, *MaEXPA15*, *MaGWD1*, and *MaLSF2*, and stimulates their transcription (Fig. 3D and E), which also validates the accuracy of the DAP-Seq data. Transient over-expression and knockdown of *MabHLH28* expression in banana fruit were able to accelerate and retard fruit softening by up- and down-regulating the expression of *MaPG3*, *MaPE1*, *MaPL5*, *MaPL8*, *MaEXP1*, *MaEXP2*, *MaEXPA2*, *MaEXPA15*, *MaGWD1*, and *MaLSF2*, respectively (Fig. 4D and H). Overexpression of *MabHLH28* in tomato fruit promoted the ripening process by upregulating the expression levels of tomato softening-related genes (Fig. 5). These findings together increased the credibility of the idea that MabHLH28 positively regulates fruit softening-related gene transcription through direct association with their promoters. It is worth noting that except for the change of fruit firmness, the ethylene production was also affected in *MabHLH28*-overexpressing and -silencing banana fruits (Fig. 4C and G). Consistent with this, using the EMSA analysis, we found that MabHLH28 can directly bind to the promoters of the ethylene biosynthesis genes *MaACS1* and *MaACO1* (Fig. S6, see online supplementary material). These observations suggest that MabHLH28 may regulate ethylene biosynthesis directly or indirectly by interacting with other TFs that target ethylene biosynthetic genes.

Interaction between TFs may have a substantial effect on the activation or repression of target gene expression, as TF binding capacity is dependent on TF concentration and/or its TF partners or co-factors [58]. It was found that bHLHs usually form homo- or heterodimers by interacting with themselves or other bHLH proteins [59]. For example, banana MabHLH1/2/4 interact with each others to make up heterodimers functioning in chilling stress [52]. In apple, MdMYC2 interacts with itself to constitute homodimer [60]. Interestingly, in grapevine, VvMYC1 alone could not activate the transcription of flavonoid pathway genes, but it modulated anthocyanin and proanthocyanin generation by forming heterodimers with MYB5a, MYB5b, MYBA1/A2, and MYBPA1 proteins [61]. In this context, MabHLH28 interacted not only with itself but also with two TFs MaWRKY49/111 (Fig. 6A–C). Importantly, interaction between MabHLH28 and MaWRKY49/111further elevated the transcription of *MaPG3*, *MaPE1*, *MaPL5*, *MaPL8*, *MaEXP1*, *MaEXP2*, *MaEXPA2*, *MaEXPA15*, *MaGWD1*, *MaLSF2*, *MaACS1*, and *MaACO1* (Fig. 6D; Fig. S5, see online supplementary material), suggesting that protein complexes such as dimers or high order complexes may have an effect on the regulation of gene expression. An interaction between two TFs that strengthens the binding of one TF to its target genes is very interesting and essential for precise regulation of gene expression, but the mechanism underlying this synergistic effect still remains to be determined. First, we performed the EMSA to test the direct binding of MaWRKY49/111 to the promoters of these softening-associated genes. We found that both MaWRKY49 and MaWRKY111 were able to bind to the promoters of these 10 target genes via the W-box binding motifs (Fig. S7, see online supplementary material). Thus, the synergistic activation of softening-associated genes may be due, at least in part, to the co-occupation of MabHLH28 and MaWRKY49/111 on the promoters of softening-associated genes. Second, we also used EMSA to determine whether the interaction between MabHLH28 and MaWRKY49/111 has an effect on MabHLH28-mediated binding capacity. We used the probes derived from the *MaPG3* and *MaGWD1* promoters, which contain only the MabHLH28 binding motif available in the DAP-Seq analysis (Fig. 3A), but not the W-box motif (the probes used in the MabHLH28 and MaWRKY49/111 binding assays are located in different regions in each promoter sequence, as shown in Text S1, see online supplementary material). In this experiment (Fig. S8, see online supplementary material), the MabHLH28 protein, but not the MaWRKY49/111 proteins, was able to bind to the MabHLH28 binding motifs in the promoters of *MaPG3* and *MaGWD1*. Interestingly, the addition of MaWRKY49 or MaWRKY111 increased the DNA-binding capacity imposed by MabHLH28 (Fig. S8, see online supplementary material), supporting another possibility that the protein complex formed by MabHLH28 and MaWRKY49/111 may enhance the MabHLH28-mediated activation of the softening-associated genes. Taken together, the mechanism by which the interaction between MabHLH28 and MaWRKY49/111 synergistically activates the transcription of softening-associated genes could be explained by the above two reasons. In addition, as previous work has revealed that MaWRKY49/111 physically interact with MabZIP21 [25], further investigations were needed to study the biochemical and genetic machinery of how MabHLH28, MaWRKY49/111, and MabZIP21 proteins act together to mediate banana fruit ripening and softening. Moreover, given the previous findings that MaMYB4 interacts with MaBRG2/3 and MaMAPK14 to undergo protein modifications [62, 63], functional connectivity between MabHLH28 and the proteins involved in protein modifications presents opportunities for stripping away MabHLH28 under post-translational regulation during fruit ripening in a future direction.

In conclusion, we propose that MabHLH28 promotes banana fruit softening via up-regulation of softening-associated genes, such as *MaPG3*, *MaPE1*, *MaPL5*, *MaPL8*, *MaEXP1*, *MaEXP2*, *MaEXPA2*, *MaEXPA15*, *MaGWD1*, and *MaLSF2*. Additionally, we identified two MabHLH28 interacting proteins, MaWRKY49 and MaWRKY111, which further enhanced MabHLH28-mediated transcriptional activation of the softening-related genes (Fig. S9, see online supplementary material). Overall, our results indicate that MabHLH28 positively regulates fruit softening by upregulating the expression of cell wall modifying and starch degrading genes either alone, or by collaborating with MaWRKY49/111 to synergistically strengthen the softening-related gene transcription, which enriches the knowledge of the regulatory network of banana fruit softening.

## Materials and methods

### Plant materials and treatments

Banana fruit (*M. acuminate* AAA group cv. Cavendish, Baxi) at commercial maturity (110–120 d after flowering) were picked from a farm at Guangzhou (Guangdong Province, China). Banana fruit were randomly separated into two lots of 180, consisting of three replicates of 60. Two postharvest treatments comprising natural ripening (air for 18 h) and ethylene-induced ripening (100 μL L^−1^ ethylene, 18 h) were conducted, according to our previous work [40]. Following treatment, fruit were held at 22°C and 90% relative humidity (RH) for up to 7 d. Samples were collected at 0 h, 2 h, 6 h, 12 h, 1 d, 3 d, 5 d, and 7 d, and the physiological indicators were recorded.

### RT-qPCR analysis

Extraction of total RNA was carried out by the procedure previously reported [40]. RT-qPCR was carried out using the GoTaq qPCR master mix kit (Promega, Madison, WI, USA) in a CFX96 Real-Time PCR Apparatus (Bio-Rad, Hercules, CA, USA) following the manufacturer’s protocol. *MaRPS4*serves as an endogenous reference gene [64]. The primer sequences used are available in Table S1 (see online supplementary material).

### Subcellular localization assay

The entire *MabHLH28* coding region minus the termination codon was inserted into pBE-GFP vector. The recombinant plasmid and the nuclear marker NLS-mCherry were transformed into the leaf cells of *N. benthamiana*. GFP and mCherry fluorescent signals were monitored via a fluorescence microscope (Axioskop 2 Plus; Zeiss, Jenna, Germany) as described by Wu *et al.* [65]. The excitation wavelengths for GFP and mCherry were 488 and 594 nm, respectively, and the emission filter wavelengths for GFP were 505–530 nm and 610–650 nm for mCherry.

### DAP-Seq analysis

DAP-Seq was conducted by the procedure previously described by Yang *et al.* [62]. The gDNA was extracted from banana fruit to construct the sequencing library. The coding sequence of *MabHLH28* was combined into thepFN19K vector to yield the affinity-purified Halo-MabHLH28 protein. Then, the Halo-MabHLH28 protein was incubated with the gDNA library. The bound DNA fragments were sequenced on an Illumina NavoSeq, and the reads were mapped to the genome sequence of *Musa*. MEME-ChIP was used to discover the core binding motif ofMabHLH28. The candidate target genes were analysed using the GO and KEGG pathway prediction.

### EMSA

The *MabHLH28* (N1–309 aa), *MaWRKY49C* (N101–300 aa), and *MaWRKY111* (N1–286 aa) coding regions were subcloned into pGEX-4 T-1 to generate and purify the recombinant GST tag proteins in accordance with the standard protocols (Clontech, Mountain View, CA, USA). The probes containing the MabHLH28-binding sites were biotinylated using a DNA 3′ End Biotinylation Kit (Thermo Scientific, Waltham, MA, USA). EMSA was conducted using a Chemiluminescent EMSA kit (Thermo Scientific, Waltham, MA, USA) as per the manufacturer’s guidelines.

### Yeast two-hybrid assay

The full-length sequence of *MabHLH28* was ligated into pGADT7 vector (AD). The full-length sequences of*MaWRKY49*, *MaWRKY111*, and *MabHLH28* were subcloned into the pGBKT7 vector (BD). Different combinations of the AD and BD constructs plasmids were transferred into the yeast strain and grown on SD/−Leu/−Trp for 3 d. Transformed colonies were plated onto SD/−Leu/−Trp/−Ade/-His, and the possible interactions were evaluated based on their growth situation and α-galactose activity according to the yeast manual handbook (Clontech, Mountain View, CA, USA).

### BiFC assay

The *MaWRKY49*, *MaWRKY111*, and *MabHLH28* coding sequences were constructed into the pSPYNE and pSPYCE vectors, respectively. The constructs were co-transformed into tobacco and YFP signal was observed (excitation at 514 nm and emission at 525–546 nm) as described previously [62].

### LCI assay

The full-length sequences of*MaWRKY49*, *MaWRKY111*, and *MabHLH28* were cloned into the pCAMBIA1300-nLUCandpCAMBIA1300-cLUC vectors, respectively. The fusion vectors were introduced into *A. tumefaciens* strain GV3101 (p19), and then co-infiltrated into *N. benthamiana* leaves in different combinations. Forty-eight hours after infiltration, the LUC fluorescent signals were measured with Luciferase Reporter Gene Assay Kit (Yeasen, Shanghai, China) using the ChemiDoc™ MP imaging equipment (Bio-Rad, Hercules, CA, USA).

### Transient expression in tobacco leaves

The coding sequence of *MabHLH28* was cloned into the pGreenII 62-SK-BD vector for transcription activity analysis. The *MabHLH28* coding region was ligated into pGreenII 62-SK vector as effector. The promoters of cell wall modifying genes (*MaPG3*, *MaPE1*, *MaPL5*, *MaPL8*, *MaEXP1*, *MaEXP2*, *MaEXPA2*, and *MaEXPA15*) and starch degradation-related genes (*MaGWD1* and *MaLSF2*) were constructed into pGreennII0800-LUC vector as reporters. As described earlier [65, 66], the constructed plasmids were infiltrated into tobacco leaves with different combinations, and then the luciferase activity was detected with Dual Luciferase Assay reagents (Yeasen, Shanghai, China) using the Luminoskan Ascent Microplate Luminometer (Thermo Fisher Scientific, Waltham, MA, USA).

### Promoter activity

The *MabHLH28* promoter was cloned into the pGreenII 0800-LUC vector and the recombinant construct was transferred into *N. benthamiana* leaves using *Agrobacterium* strain EHA105 (pSoup). The transformed *N. benthamiana* leaves were treated with or without ethylene (100 μL L^−1^), and the *Renilla* and firefly luciferase activities were measured with Dual Luciferase Assay reagents (Yeasen, Shanghai, China) using the Luminoskan Ascent Microplate Luminometer (Thermo Fisher Scientific, Waltham, MA, USA) as previously described [67].

### Transient expression in banana fruit

The coding sequence of *MabHLH28* was subcloned into the pCXUN-HA vector. The fusion construct was transformed into *A. tumefaciens* strain EHA105 and then injected into banana fruits as described by Shan *et al.* [67]. The infiltrated banana was ripened with 100 μL L^−1^ ethylene, and then maintained at 22°C and 90% RH for up to 5 d. Samples were taken at days 0, 1, 2, 3, 4, and 5, and relevant physiological indicators were measured.

To silence the expression of *MabHLH28* in banana fruit, a partial MabHLH28 (1–300 bp) was ligated into the pTRV2 vector (http://vigs.solgenomics.net/). The recombinant plasmid was introduced in *A. tumefaciens* strain EHA105, and VIGS of*MabHLH28* in banana fruit was performed according to our previous method [11]. The infiltrated banana fruit were subjected to 100 μL L^−1^ ethylene treatment and held at 22°C and 90% RH for up to 7 d. The relevant physiological indexes and gene expression were detected at days 0, 1, 3, 5, and 7.

### Tomato genetic transformation analysis

The full-length of MabHLH28 was ligated into the pBI121-FLAG plasmid. Subsequently, the resulting vector was introduced into the *A. tumefaciens* strain GV3101. *Agrobacterium*-mediated transformation was performed using the leaf disks derived from the tomato ‘Micro-Tom’, as described by Fan *et al.* [68]. Transgenic plants were screened by the transcript and translational levels of MabHLH28, and three independently transgenic lines (T2) were available for experiments. The wild-type and transgenic tomato fruits at 24, 29, 32, and 35 DPA were collected pending use.

### Antibody and gene accessions

The anti-GST (Abcam, Cambridge, MA, USA; Cat. No. ab9085), anti-HA (Sigma-Aldrich, Steinheim, Germany; Cat. No. H6908) and anti-FLAG (Sigma–Aldrich, Steinheim, Germany; Cat. No. F1804) antibodies were used in this study. Sequence data from this article can be found in the genome of banana and tomato as the following accessions: *MabHLH28* (Ma02_g11480), *MaWRKY49* (Ma04_g24790), *MaWRKY111* (Ma07_g26230), *MaPG3* (Ma02_g04450), *MaPE1* (Ma07_g11280), *MaPL5* (Ma06_g30000), *MaPL8* (Ma07_g04670), *MaEXP1* (Ma05_g07230), *MaEXP2* (Ma11_g21280), *MaEXPA2* (Ma05_g07240), *MaEXPA15* (Ma06_g12190), *MaGWD1* (Ma03_g15660), *MaLSF2* (Ma04_g00190), *SlPG2* (Solyc10g080210), *SlPL* (Solyc03g111690), *SlPE3* (Solyc07g064190), *SlEXP1* (Solyc06g051800), *SlGWD1* (Solyc05g005020), *SlLSF2* (Solyc06g050230).

### Statistics

All experiments were repeated with at least three replicates. ANOVA were statistically analyzed using the SPSS 19.0 software. Student’s *t*-test was utilized to determine the statistical difference between samples (^*^P < 0.05 or ^**^P < 0.01).

## Acknowledgements

This work was financially supported by the National Key R & D Program of China (2022YFD2100102), China Postdoctoral Science Foundation (2023 M731146), Innovative Team Project of Guangdong Universities (2022KCXTD051), and China Agriculture Research System of MOF and MARA (CARS-31).

## Author contributions

J.K. and X.S. conceived the research and designed the experiments; C.W. performed most of the experiments and analysed the data; D.C., J.L., Z.L., and W.W. performed some of the experiments; J.K., C.W., and X.S. wrote the manuscript; W.S., J.C., and W.L. gave advice and revised the manuscript. All authors read and approved the final manuscript.

## Data availability

All data from this study are included in the submitted article.

## Conflict of interest statement

The authors declare that they have no competing interests.

## Supplementary data

Supplementary data is available at *Horticulture Research* online.

## Supplementary Material

Web_Material_uhae053
