# Supplementary material for: Banana MabHLH28 positively regulates the expression of softening-related genes to mediate fruit ripening independently or via cooperating with MaWRKY49/111
Source: Hortic Res. 2024 Feb 23;11(4):uhae053. doi: 10.1093/hr/uhae053 (PMC11069428; doi:10.1093/hr/uhae053)
Supplement: Web_Material_uhae053 [file web_material_uhae053.docx]

**Supplementary data**

**
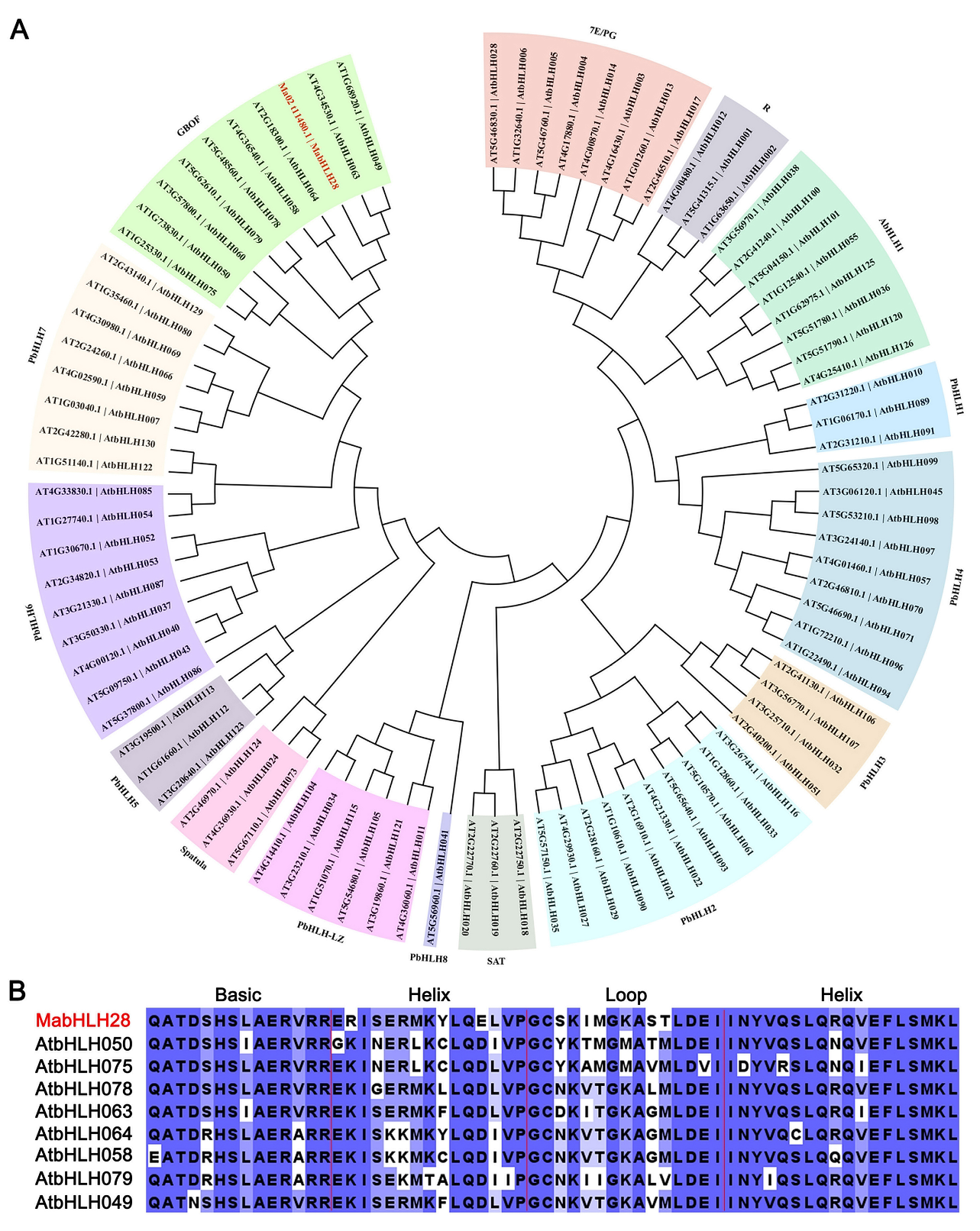
**

**Fig. S1.** Phylogenetic relationship and multiple sequence comparison analysis of MabHLH28. (**A**) Phylogenetic relationship analysis of MabHLH28 with the bHLH proteins from Arabidopsis. Multiple alignment was carried out using CLUSTALW and the phylogenetic tree was constructed with MEGA-Ⅹ using a bootstrap test of phylogeny with neighbor-joining method. Bootstrapping with 1,000 replicates was used to assess the statistical reliability of nodes in the tree. (**B**) Multiple sequence comparison analysis of MabHLH28 with the bHLH proteins in Arabidopsis from the same subgroup.

**
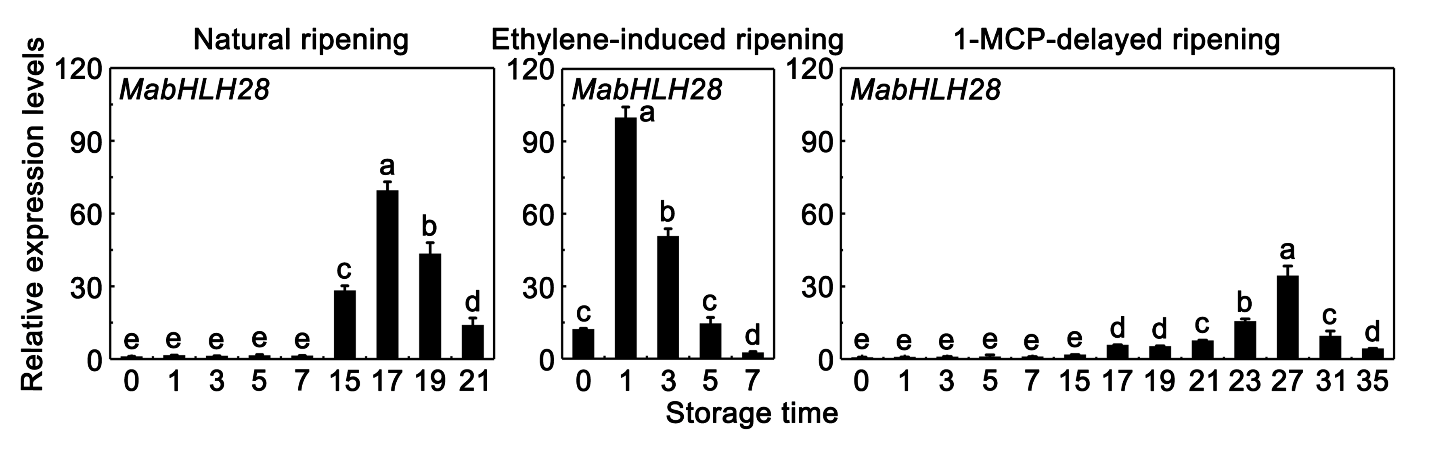
**

**Fig. S2.** Expression of *MabHLH28* in banana fruit with three different ripening pattens consisting of natural, ethylene-induced, and 1-MCP-delayed ripening as reported in [25]. Each value is the mean ± SE of three replicates. Different letters indicate significant differences at *p* < 0.05 level.

**
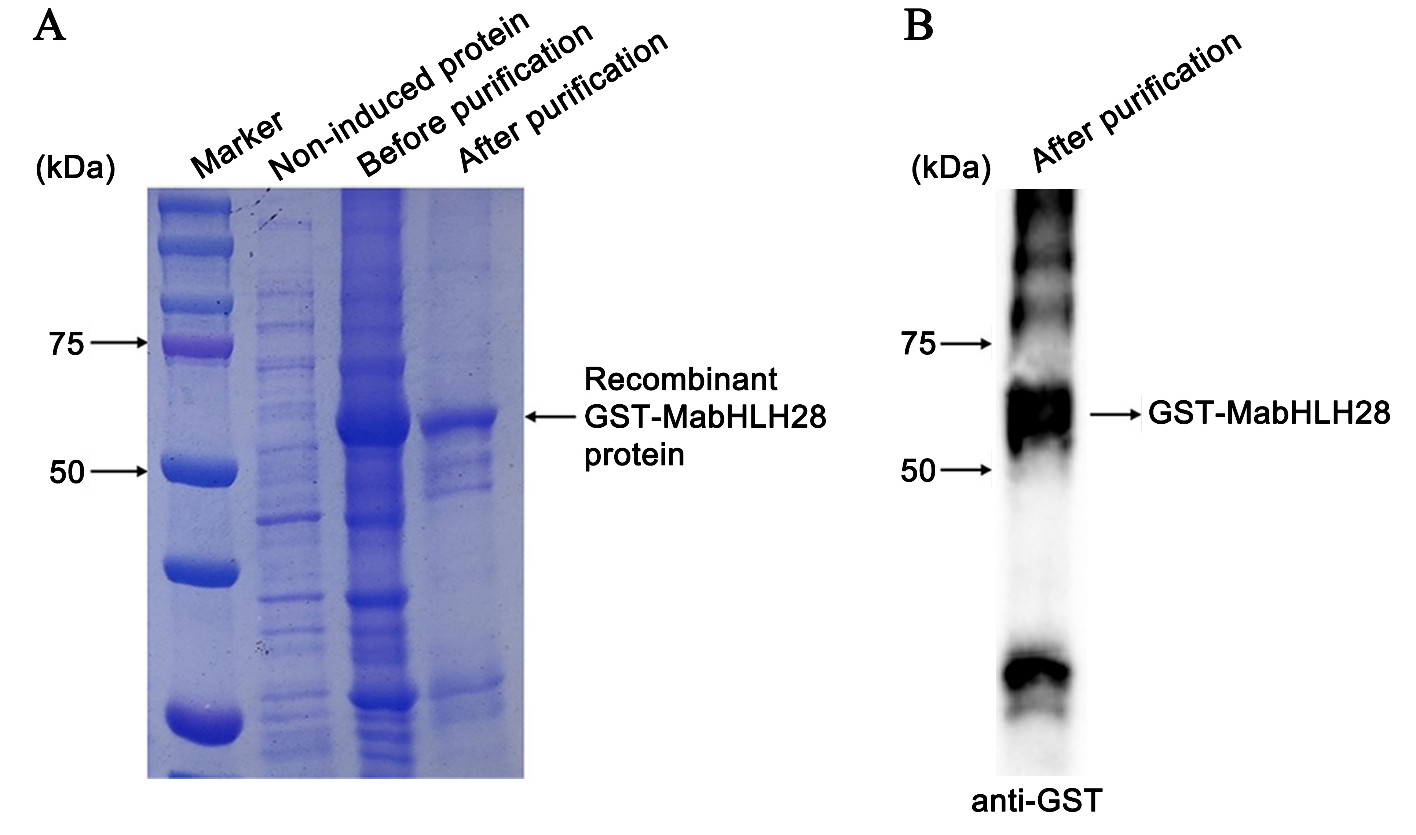
**

**Fig. S3.** Recombinant purified GST-MabHLH28 protein. (**A**) SDS-PAGE gel stained with Coomassie brilliant blue demonstrating affinity purification of the recombinant GST-tagged MabHLH28 protein used for study. (**B**) Western blot analysis of the s the exact location of recombinant GST-MabHLH28 protein.

**
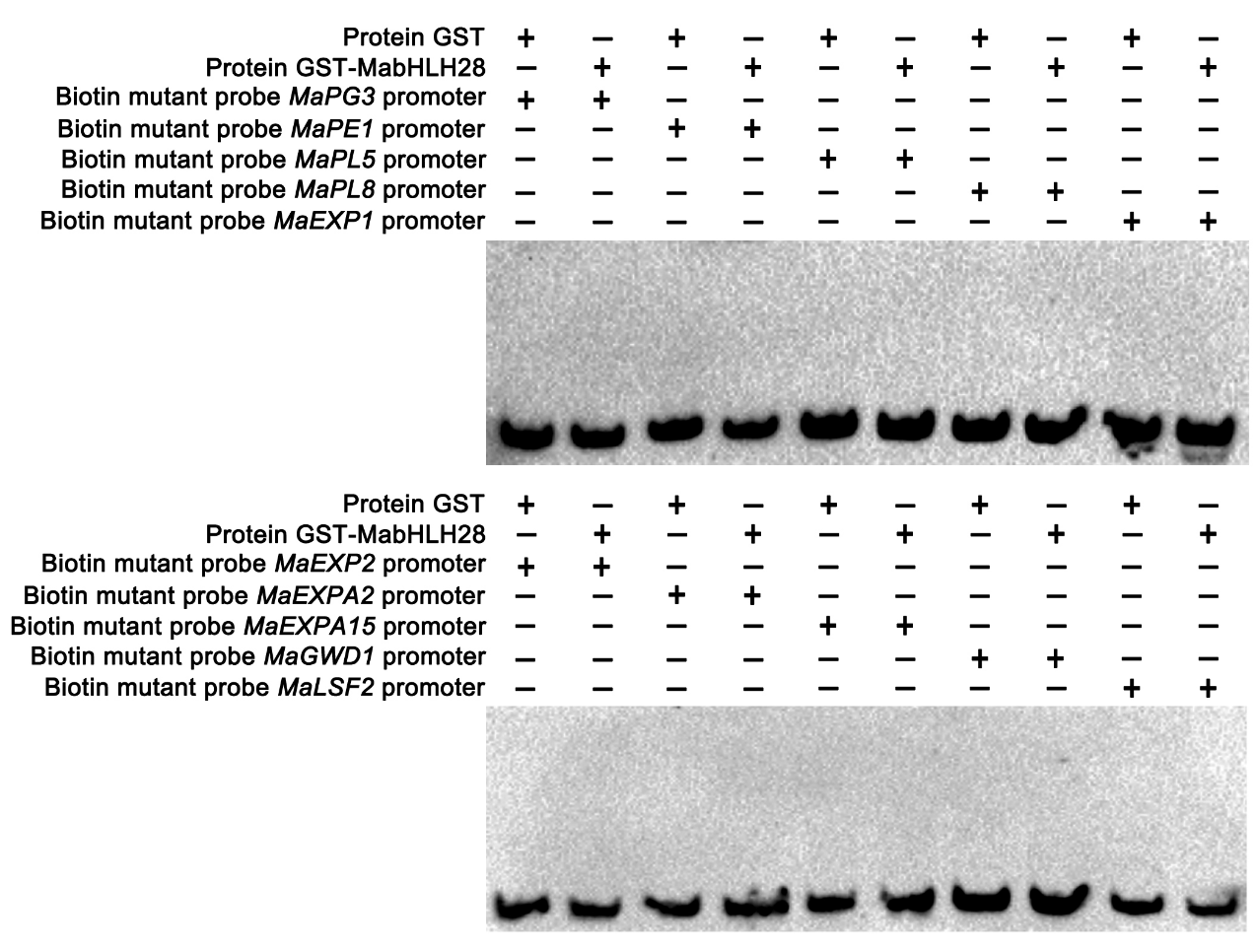
**

**Fig. S4.** The GST-MabHLH28 protein cannot bind efficiently to the promoters of softening-related genes (*MaPG3*, *MaPE1*, *MaPL5*, *MaPL8*, *MaEXP1*, *MaEXP2*, *MaEXPA2*, *MaEXPA15*, *MaGWD1*, and *MaLSF2*) with mutated MabHLH28-binding motifs. − and + represent absence or presence.

**
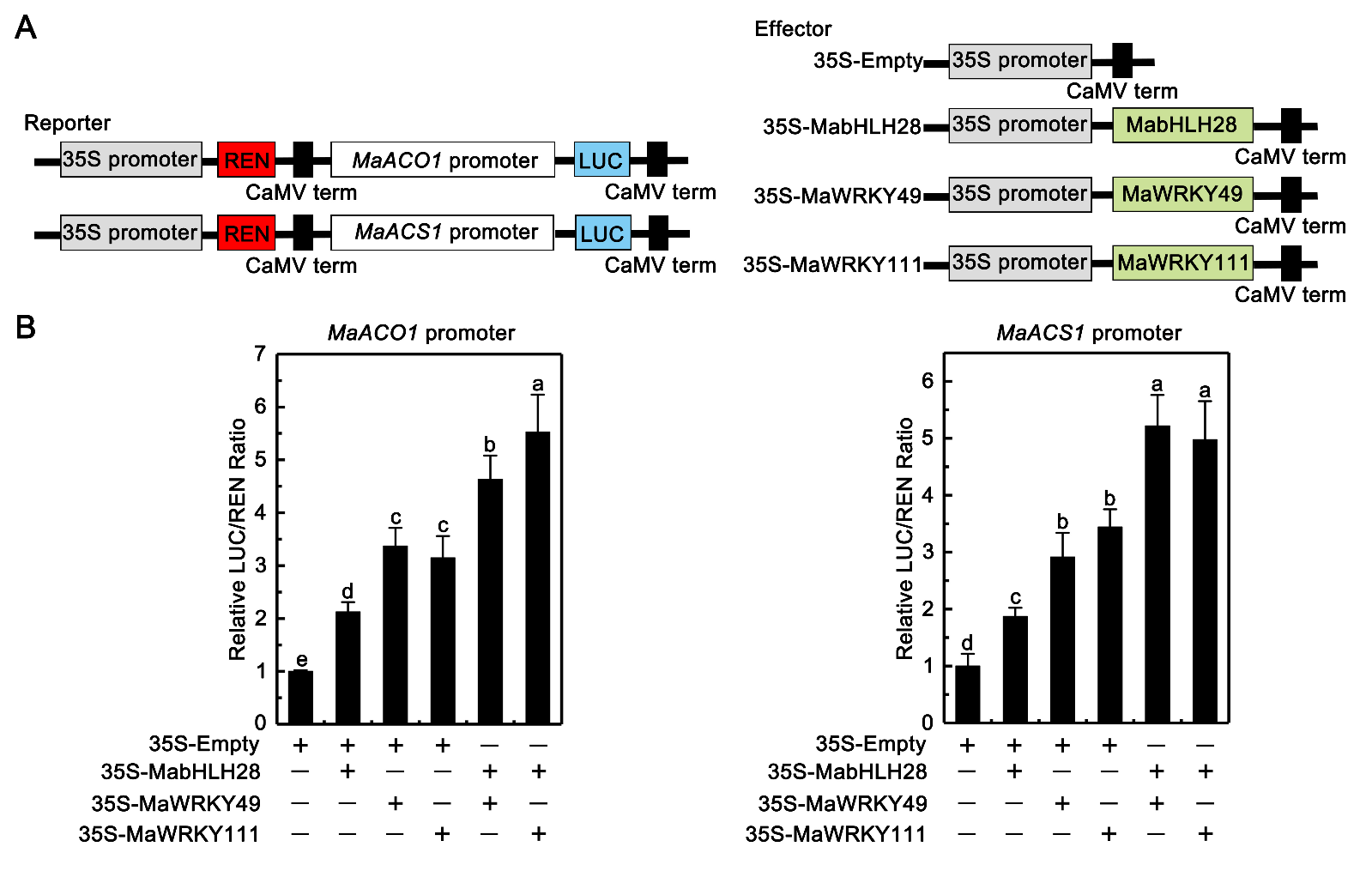
**

**Fig. S5.** Interaction between MabHLH28 and MaWRKY49/111 synergistically enhanced the transcription of *MaACS1* and *MaACO1*. (**A**) Schematic program of the reporter and effector constructs used in the dual-luciferase reporter assay. (**B**) DLR assay showing that association between MabHLH28 and MaWRKY49/111 further elevated the transcription of *MaACS1* and *MaACO1*. The ratio of LUC/REN of the empty 62-SK vector (negative control) was set to 1. Each value as mean ± SE of six replicates. Different letters denote significant difference computed (*P* < 0.05).


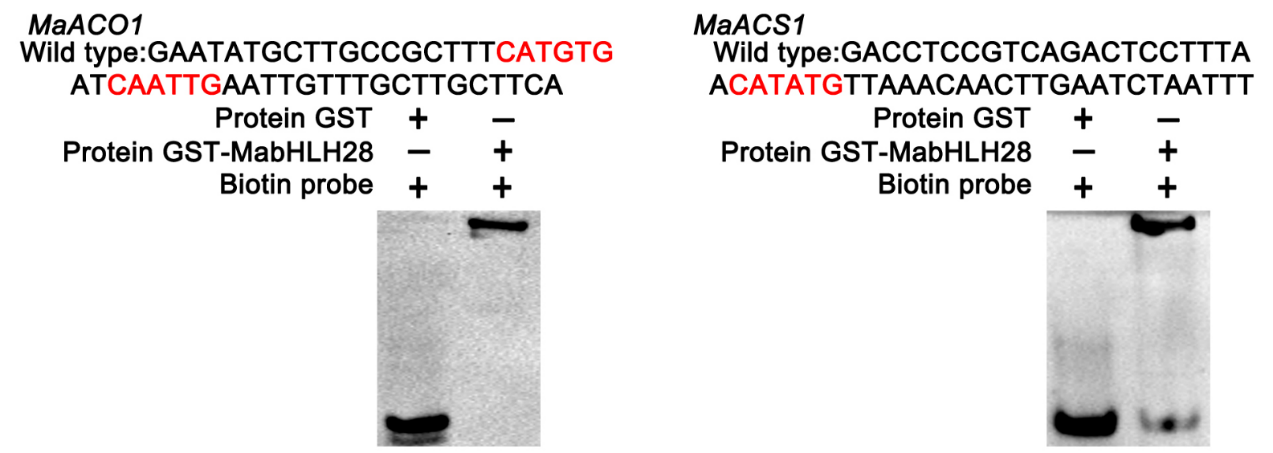


**Fig. S6.** EMSA assays showed that MabHLH28 binds to the promoters of ethylene biosynthetic genes *MaACO1* and *MaACS1*. The MabHLH28-binding motifs are marked by red color. − and + represent absence or presence.


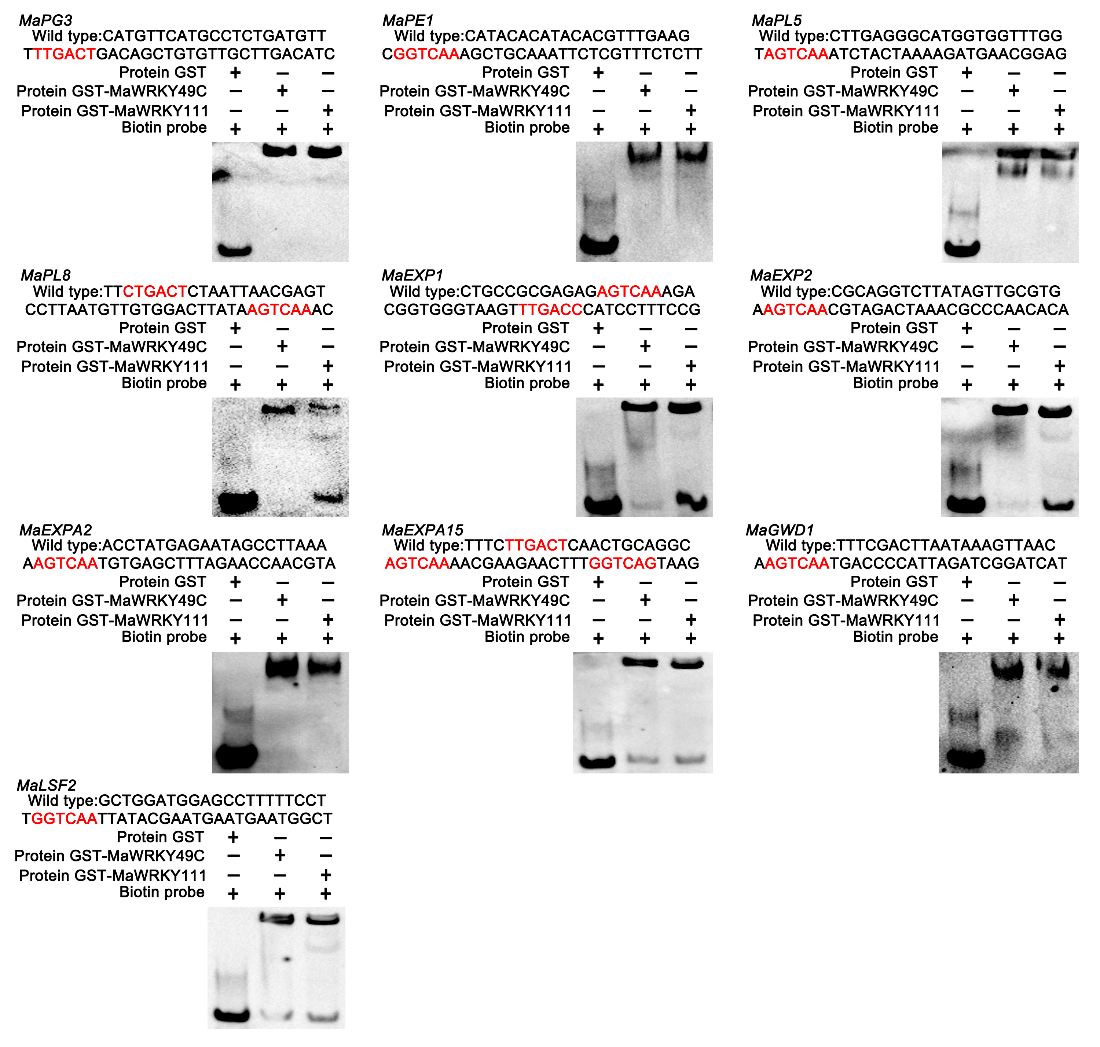


**Fig. S7.** EMSA assays showed that MaWRKY49 and MaWRKY111 interact with the promoters of softening-related genes (*MaPG3*, *MaPE1*, *MaPL5*, *MaPL8*, *MaEXP1*, *MaEXP2*, *MaEXPA2*, *MaEXPA15*, *MaGWD1*, and *MaLSF2*). The W-box motifs are indicated in red color. − and + represent absence or presence.


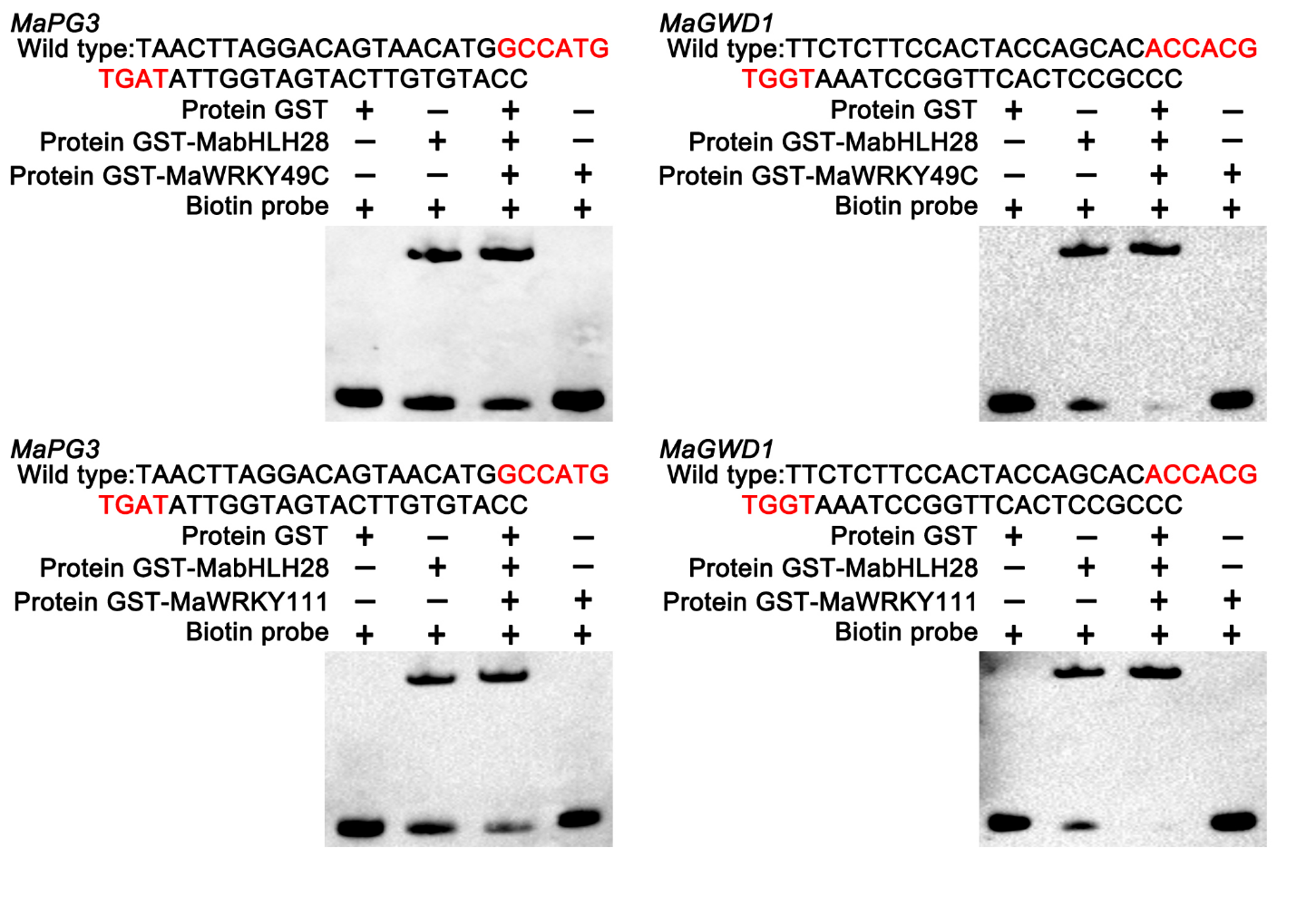


**Fig. S8.** EMSA showing the interaction between MabHLH28 and MaWRKY49/111 increases binding affinity of MabHLH28. The MabHLH28-binding motifs are illustrated in red color. − and + represent absence or presence.


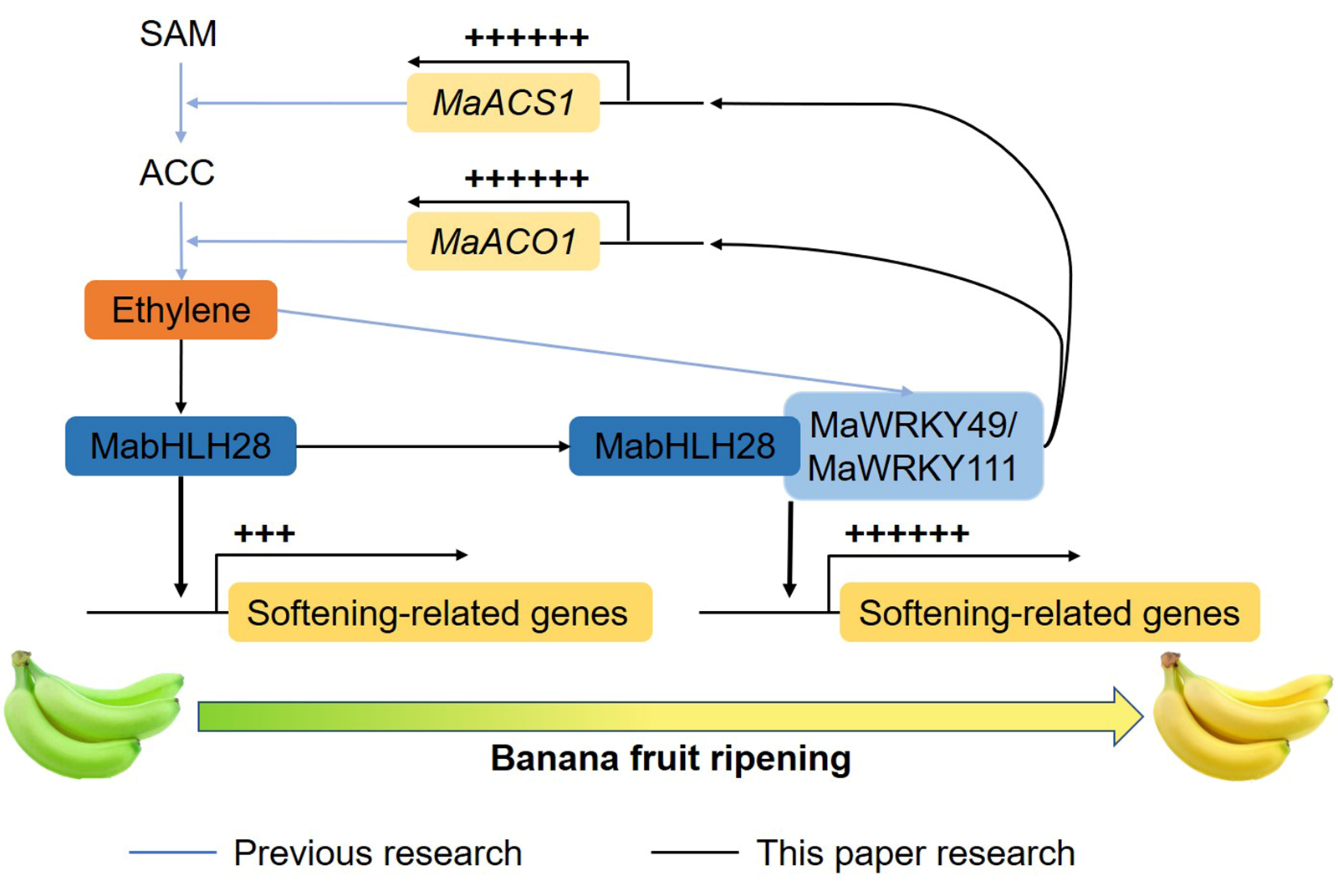


**Fig. S9** A postulated model of MabHLH28 alone or in combination with MaWRKY49/111 in controlling banana fruit softening. Ethylene treatment induces the expression of *MabHLH28*, and MabHLH28 binds directly to and activates the transcription of a subset of softening-related genes to mediate fruit ripening. Importantly, MabHLH28 interacts with two TFs MaWRKY49/111, which function as transcriptional activators of the ethylene biosynthetic genes *MaACS1* and *MaACO1* [25]. The MabHLH28-MaWRKY49/111 protein complex results in further activation of ripening-associated genes to promote the ripening of banana. SAM, S-adenosyl-L-methionine; ACC, 1-aminocyclopropane-1-carboxylic acid. The arrows in black colour represent the findings available in this study, while the arrows in weak blue colour denote the findings previously reported [25].

**Table S1** Summary of primers used in this study.

| **Assay** | **Primer sequence (5’-3’)** | | **Restriction site** |
| --- | --- | --- | --- |
| **RT-qPCR** | ***MaRPS4-qF: TGAGAGTGGCTTGACCCTGA***  ***MabHLH28-qF:*** ***GCCTGGATGCAGTAAGATCATG***  ***MaPG3-qF: TCGGTATCTGATCGAGCTTTGG***  ***MaPE1-qF: CGGTGATAATGGAGTCGGAGA***  ***MaPL5-qF: AGATATACGTGGTGACCGACAG***  ***MaPL8-qF:*** ***GCTTTTGGGTCACAGTGATTCT***  ***MaEXP1-qF: TTGCTCACTCACGACTTCAATC***  ***MaEXP2-qF: TCTGGGAGATGATTGGATTCCT***  ***MaEXPA2-qF: CTTTCTCCAGATAGCGCAGTAC***  ***MaEXPA15-qF:*** ***CAGCATTTCGATCTCTCTCAGC***  ***MaGWD1-qF: AGACTTCCCACAACATAGAG***  ***MaLSF2-qF: AATGGGCTATTTCTGAGGGTAA***  ***SlActin-qF: TGTCCCTATTTACGAGGGTTATGC***  ***SlPG2-qF: TTCAGGTCCATGCAGATCTTCT***  ***SlPL-qF: ACTGTGGATTATTTTCGCGAGG***  ***SlPE3-qF: GGCTAAGTGGTGTGCTTACAAA***  ***SlEXP1-qF: ATCACAGCTACCAATTTCTGCC***  ***SlGWD1-qF: GTGGGAGAAAGCAGGAAAGC***  ***SlLSF2-qF: CCCCTGCTGTTTCAATTGCT*** | ***MaRPS4-qR: GTGACATTTAGTCGTCTGCTGG***  ***MabHLH28-qR:*** ***GAACACTACTGGAATTGCAGGG***  ***MaPG3-qR: CGTCGGATGTGAAATGGACAAT***  ***MaPE1-qR: CACTTGTGATCACCTTGTACCC***  ***MaPL5-qR: GGTCTTGTGGGAGTTCATGATG***  ***MaPL8-qR:*** ***TGTTGATGGTTGGATTTGCACT***  ***MaEXP1-qR: TATGAAGGAGCTGAAGGAGGAC***  ***MaEXP2-qR:*** ***CGTCGGTGTACAAGTTCTGATA***  ***MaEXPA2-qR:*** ***AACCTTATGCCTCCCTTCTTCA***  ***MaEXPA15-qR:*** ***TCAGAACCAGGTTGAAGTAGGA***  ***MaGWD1-qR: AAGTGCCTGACAGATTACGA***  ***MaLSF2-qR: GCTCTTTCCACGGGTCATTCTT***  ***SlActin-qR: CAGTTAAATCACGACCAGCAAGAT***  ***SlPG2-qR: CACCATACTTGTCCATTGCCAT***  ***SlPL-qR: CACCATAGCATTTCCACCTTGT***  ***SlPE3-qR: CCCTGCCTCAAAACTTCATCAT***  ***SlEXP1-qR: TGGGATCCTGCGATAAGTTACA***  ***SlGWD1-qR: AAGCTGCCCAAAGTCTCTCT***  ***SlLSF2-qR: CTGGCAGATTCTCAAAGGGC*** |  |
| **Full length cloning** | ***MabHLH28-F: ATGCAGTGCCCAACAGCTCCTC***  ***MabHLH28-R: TCACTGACCTCGGCAATTGGTACC*** | |  |
| **Y2H assay** | ***MabHLH28-AD-F: ggaggccagtgaattc ATGCAGTGCCCAACAGCTCCTC***  ***MabHLH28-AD-R: cgagctcgatggatcc TCACTGACCTCGGCAATTGGTACC***  ***MabHLH28-BD-F: catggaggccgaattc ATGCAGTGCCCAACAGCTCCTC***  ***MabHLH28-BD-R: gccgctgcaggtcgacg TCACTGACCTCGGCAATTGGTACC***  ***MaWRKY49-BD-F: catggaggccgaattc ATGGGCGACGATAACTGGGATCTT***  ***MaWRKY49-BD-R: gccgctgcaggtcgacg TTAGATCGCCATGTCTGCCGCC***  ***MaWRKY111-BD-F: catggaggccgaattc ATGAACGGGAGCTGCAGCGACG***  ***MaWRKY111-BD-R: gccgctgcaggtcgacg TCACCCGGTGGTCCCCACC*** | | ***EcoR* Ⅰ**  ***BamH* I**  ***EcoR* Ⅰ**  ***Sal* Ⅰ**  ***EcoR* Ⅰ**  ***Sal* Ⅰ**  ***EcoR* Ⅰ**  ***Sal* Ⅰ** |
| **Subcellular localization** | ***MabHLH28-pBE-GFP-F:*** ***atctagagcagtcgacggtacc ATGCAGTGCCCAACAGCTCCTC***  ***MabHLH28-pBE-GFP-R:*** ***ctcctcgcccttgctcaccat CTGACCTCGGCAATTGGTACC*** | | ***Kpn* I**  ***BamH* I** |
| **BiFC assay** | ***MabHLH28-YNE-F: ggcgcgccactagtggatcc*** ***ATGCAGTGCCCAACAGCTCCTC***  ***MabHLH28-YNE-R:*** ***gcggtaccctcgaggtcgac*** ***CTGACCTCGGCAATTGGTACC***  ***MabHLH28-YCE-F: ggcgcgccactagtggatcc ATGCAGTGCCCAACAGCTCCTC***  ***MabHLH28-YCE-R:*** ***gcggtaccctcgaggtcgac CTGACCTCGGCAATTGGTACC***  ***MaWRKY49-YCE-F: ggcgcgccactagtggatcc ATGGGCGACGATAACTGGGATCTT***  ***MaWRKY49-YCE-R:*** ***gcggtaccctcgaggtcgac GATCGCCATGTCTGCCGCC***  ***MaWRKY111-YCE-F: ggcgcgccactagtggatcc ATGAACGGGAGCTGCAGCGACG***  ***MaWRKY111-YCE-R: gcggtaccctcgaggtcgac CCCGGTGGTCCCCACC*** | | ***BamH* I**  ***Sal* I**  ***BamH* I**  ***Sal* I**  ***BamH* I**  ***Sal* I**  ***BamH* I**  ***Sal* I** |
| **Promoter islation** | ***MaPG3-pro-F: ACATAATCATGGCGATAAAGGAGCCTG***  ***MaPG3-pro-R: CGTTATCAGCTATGATCTCCTCAGA***  ***MaPE1-pro-F: ATTGATCTTTCCTGAATTATGAATCCCTTGA***  ***MaPE1-pro-R: CATGTTCGAGCCGAATTCCTTAGG***  ***MaPL5-pro-F: TGTCGTGATCGACGGCCCACATA***  ***MaPL5-pro-R: TTTTGTTCTCCGGTAAGTTGAGAGTTGT***  ***MaPL8-pro-F: CTTCTTGAGGATAAGAACACCTACCG***  ***MaPL8-pro-R: CGTCGCCCTCTTCGCCTCTCT***  ***MaEXP1-pro-F: GCGGGCTAGTCTTAGGCTGGA***  ***MaEXP1-pro-R: AGTTGAGAGTGAGGGTTGGAGTTGGTTT***  ***MaEXP2-pro-F: AGGAGCCTCTTGCAAGCGACAC***  ***MaEXP2-pro-R: CTCTACTTGCTCGAAGAAGACAACT***  ***MaEXPA2-pro-F: TCTTTGATACATTCTCACAAGTTGGGAA***  ***MaEXPA2-pro-R: CGGAGCCGTGGGTGAGCAAG***  ***MaEXPA15-pro-F: GCTTTCGCCATCCATCCCCCA***  ***MaEXPA15-pro-R: CCTCCTGCACCAAAAGAAATGGTTAT***  ***MaGWD1-pro-F: CCGCCACCCCGTTATCACC***  ***MaGWD1-pro-R: GAGTGACTCGCGGGAGAGAGAG***  ***MaLSF2-pro-F: CCGTCCGGGTTCCTTGAGG***  ***MaLSF2-pro-R: CCCCGCCTCTTCCTTCTCTC***  ***MabHLH28-pro-F: GTCACTCAAATCTTCCTTGTCTCCT***  ***MabHLH28-pro-R: GGTCTTCTCTTATCTATGGATCTT*** | |  |
| **Protein expression and EMSA assay** | ***MabHLH28-GST-F: ggttccgcgtggatcc*** ***ATGCAGTGCCCAACAGCTCCTC***  ***MabHLH28-GST-R: agtcacgatgcggccgc TCACTGACCTCGGCAATTGGTACC***  ***MaWRKY49C-GST-F: ggttccgcgtggatcc ATGCGTCCGGTCTCCCAAGCTC***  ***MaWRKY49C-GST-R: agtcacgatgcggccgc TTAGATCGCCATGTCTGCCGCC***  ***MaWRKY111-GST-F: ggttccgcgtggatcc ATGAACGGGAGCTGCAGCGACG***  ***MabHLH111-GST-R: agtcacgatgcggccgc TCACCCGGTGGTCCCCACC***  ***MaPG3-probe-F: TAACTTAGGACAGTAACATGGCCATGTGATATTGGTAGTACTTGTGTACC***  ***MaPG3-probe-R: GGTACACAAGTACTACCAATATCACATGGCCATGTTACTGTCCTAAGTTA***  ***MaPG3-mprobe-F: TAACTTAGGACAGTAACATGAAAAAAAAAAATTGGTAGTACTTGTGTACC***  ***MaPG3-mprobe-R: GGTACACAAGTACTACCAATTTTTTTTTTTCATGTTACTGTCCTAAGTTA***  ***MaPE1-probe-F: TCAGCGTTCACATGCATGCGTCGTTTGTCATGAAACCACGTGGATGTATA***  ***MaPE1-probe-R: TATACATCCACGTGGTTTCATGACAAACGACGCATGCATGTGAACGCTGA***  ***MaPE1-mprobe-F: TCAGCGAAAAAAAAAATGCGTCGTTTGTCATGAAAAAAAAAAAATGTATA***  ***MaPE1-mprobe-R: TATACATTTTTTTTTTTTCATGACAAACGACGCATTTTTTTTTTCGCTGA***  ***MaPL5-probe-F: GCTCTGCGGCCGAAAGCCATCCCATGTGCTGTGCAGTGCCGTGAACACCC***  ***MaPL5-probe-R: GGGTGTTCACGGCACTGCACAGCACATGGGATGGCTTTCGGCCGCAGAGC***  ***MaPL5-mprobe-F: GCTCTGCGGCCGAAAGCCATAAAAAAAAAAGTGCAGTGCCGTGAACACCC***  ***MaPL5-mprobe-R: GGGTGTTCACGGCACTGCACTTTTTTTTTTATGGCTTTCGGCCGCAGAGC***  ***MaPL8-probe-F: GAGGATTTGGCGCACGAGGTCATGTTGCATGCATGTGCAAACCTACTGCT***  ***MaPL8-probe-R: AGCAGTAGGTTTGCACATGCATGCAACATGACCTCGTGCGCCAAATCCTC***  ***MaPL8-mprobe-F: GAGGATTTGGAAAAAAAAAACATGTTGCAAAAAAAAAAAAACCTACTGCT***  ***MaPL8-mprobe-R: AGCAGTAGGTTTTTTTTTTTTTGCAACATGTTTTTTTTTTCCAAATCCTC***  ***MaEXP1-probe-F: GGCAGCCCGGAATAGCATGTGCTCCTCCTCACATGGTGGGACAGTCCGTA***  ***MaEXP1-probe-R: TACGGACTGTCCCACCATGTGAGGAGGAGCACATGCTATTCCGGGCTGCC***  ***MaEXP1-mprobe-F: GGCAGCCCGGAATAAAAAAAAAACCTCAAAAAAAAAAGGGACAGTCCGTA***  ***MaEXP1-mprobe-R: TACGGACTGTCCCTTTTTTTTTTGAGGTTTTTTTTTTATTCCGGGCTGCC***  ***MaEXP2-probe-F: AAGTCTCACCTCTCACGCCACGCACGAGTCACAAGTCATCTCCCTTTCTG***  ***MaEXP2-probe-R: CAGAAAGGGAGATGACTTGTGACTCGTGCGTGGCGTGAGAGGTGAGACTT***  ***MaEXP2-mprobe-F: AAGTCTCACCTCTCACGCCAAAAAAAAAAAACAAGTCATCTCCCTTTCTG***  ***MaEXA2-mprobe-R: CAGAAAGGGAGATGACTTGTTTTTTTTTTTTGGCGTGAGAGGTGAGACTT***  ***MaEXPA2-probe-F: TGGCAATTTGGAAGTGCTGACACACGTGTTCATTATTAGACTCATTGTTT***  ***MaEXPA2-probe-R: AAACAATGAGTCTAATAATGAACACGTGTGTCAGCACTTCCAAATTGCCA***  ***MaEXPA2-mprobe-F: TGGCAATTTGGAAGTGCTGAAAAAAAAAAACATTATTAGACTCATTGTTT***  ***MaEXPA2-mprobe-R: AAACAATGAGTCTAATAATGTTTTTTTTTTTCAGCACTTCCAAATTGCCA***  ***MaEXPA15-probe-F: GCTTTTGTTACCGCGGCATGTGGCTTCCCATGTGCGGTGGGTCGACGCAT***  ***MaEXPA15-probe-R: ATGCGTCGACCCACCGCACATGGGAAGCCACATGCCGCGGTAACAAAAGC***  ***MaEXPA15-mprobe-F: GCTTTTGTTACCGCAAAAAAAAAATTAAAAAAAAAAGTGGGTCGACGCAT***  ***MaEXPA15-mprobe-R: ATGCGTCGACCCACTTTTTTTTTTAATTTTTTTTTTGCGGTAACAAAAGC***  ***MaGWD1-probe-F: TTCTCTTCCACTACCAGCACACCACGTGGTAAATCCGGTTCACTCCGCCC***  ***MaGWD1-probe-R: GGGCGGAGTGAACCGGATTTACCACGTGGTGTGCTGGTAGTGGAAGAGAA***  ***MaGWD1-mprobe-F: TTCTCTTCCACTACCAGCACAAAAAAAAAAAAATCCGGTTCACTCCGCCC***  ***MaGWD1-mprobe-R: GGGCGGAGTGAACCGGATTTTTTTTTTTTTGTGCTGGTAGTGGAAGAGAA***  ***MaLSF2-probe-F: TATCACGTGCGGGTTAAGCCCGAAATATTAGAACTCGACGACATGTGCTC***  ***MaLSF2-probe-R: GAGCACATGTCGTCGAGTTCTAATATTTCGGGCTTAACCCGCACGTGATA***  ***MaLSF2-mprobe-F: TAAAAAAAAAAGGTTAAGCCCGAAATATTAGAACTCGACAAAAAAAAAAC***  ***MaLSF2-mprobe-R: GTTTTTTTTTTGTCGAGTTCTAATATTTCGGGCTTAACCTTTTTTTTTTA***  ***MaACO1-probe-F:*** ***GAATATGCTTGCCGCTTTCATGTGATCAATTGAATTGTTTGCTTGCTTCA***  ***MaACO1-probe-R: TGAAGCAAGCAAACAATTCAATTGATCACATGAAAGCGGCAAGCATATTC***  ***MaACS1-probe-F:*** ***GACCTCCGTCAGACTCCTTTAACATATGTTAAACAACTTGAATCTAATTT***  ***MaACS1-probe-R:*** ***AAATTAGATTCAAGTTGTTTAACATATGTTAAAGGAGTCTGACGGAGGTC***  ***MaPG3-probe-F: CATGTTCATGCCTCTGATGTTTTTGACTGACAGCTGTGTTGCTTGACATC***  ***MaPG3-probe-R: GATGTCAAGCAACACAGCTGTCAGTCAAAAACATCAGAGGCATGAACATG***  ***MaPE1-probe-F: CATACACATACACGTTTGAAGCGGTCAAAGCTGCAAATTCTCGTTTCTCTT***  ***MaPE1-probe-R: AAGAGAAACGAGAATTTGCAGCTTTGACCGCTTCAAACGTGTATGTGTATG***  ***MaPL5-probe-F: CTTGAGGGCATGGTGGTTTGGTAGTCAAATCTACTAAAAGATGAACGGAG***  ***MaPL5-probe-R: CTCCGTTCATCTTTTAGTAGATTTGACTACCAAACCACCATGCCCTCAAG***  ***MaPL8-probe-F: TTCTGACTCTAATTAACGAGTCCTTAATGTTGTGGACTTATAAGTCAAAC***  ***MaPL8-probe-R: GTTTGACTTATAAGTCCACAACATTAAGGACTCGTTAATTAGAGTCAGAA***  ***MaEXP1-probe-F: CTGCCGCGAGAGAGTCAAAGACGGTGGGTAAGTTTGACCCATCCTTTCCG***  ***MaEXP1-probe-R: CGGAAAGGATGGGTCAAACTTACCCACCGTCTTTGACTCTCTCGCGGCAG***  ***MaEXP2-probe-F: CGCAGGTCTTATAGTTGCGTGAAGTCAACGTAGACTAAACGCCCAACACA***  ***MaEXP2-probe-R: TGTGTTGGGCGTTTAGTCTACGTTGACTTCACGCAACTATAAGACCTGCG***  ***MaEXPA2-probe-F: ACCTATGAGAATAGCCTTAAAAAGTCAATGTGAGCTTTAGAACCAACGT***  ***MaEXPA2-probe-R: TACGTTGGTTCTAAAGCTCACATTGACTTTTTAAGGCTATTCTCATAGGT***  ***MaEXPA15-probe-F: TTTCTTGACTCAACTGCAGGCAGTCAAAACGAAGAACTTTGGTCAGTAAG***  ***MaEXPA15-probe-R: CTTACTGACCAAAGTTCTTCGTTTTGACTGCCTGCAGTTGAGTCAAGAAA***  ***MaLSF2-probe-F: GCTGGATGGAGCCTTTTTCCTTGGTCAATTATACGAATGAATGAATGGCT***  ***MaLSF2-probe-R: AGCCATTCATTCATTCGTATAATTGACCAAGGAAAAAGGCTCCATCCAGC*** | | ***BamH* Ⅰ**  ***Not* Ⅰ**  ***BamH* Ⅰ**  ***Not* Ⅰ**  ***BamH* Ⅰ**  ***Not* Ⅰ** |
| **Dual-luciferase expression assay** | ***MabHLH28-62SK-BD-F: cgccgtctagaactagtggatcc ATGCAGTGCCCAACAGCTCCTC***  ***MabHLH28-62SK-BD-R: tcgataagcttgatatcgaattc TCACTGACCTCGGCAATTGGTACC***  ***MabHLH28-62SK-F: ggccgctctagaactagtggatcc ATGCAGTGCCCAACAGCTCCTC***  ***MabHLH28-62SK-R: atcgataagcttgatatcgaattc CTGACCTCGGCAATTGGTACC***  ***MaWRKY49-62SK-F: ggccgctctagaactagtggatcc*** ***ATGGGCGACGATAACTGGGATCTT***  ***MaWRKY49-62SK-R: atcgataagcttgatatcgaattc TTAGATCGCCATGTCTGCCGCC***  ***MaWRKY111-62SK-F: ggccgctctagaactagtggatcc*** ***ATGAACGGGAGCTGCAGCGACG***  ***MaWRKY111-62SK-R: atcgataagcttgatatcgaattc TCACCCGGTGGTCCCCACC***  ***MabHLH28-pro-0800-F: cactatagggcgaattgggtacc GTCACTCAAATCTTCCTTGTCTCCT***  ***MabHLH28-pro-0800-R: tttatgtttttggcgtcttccat GGTCTTCTCTTATCTATGGATCTT***  ***MaPG3-pro-0800-F: cactatagggcgaattgggtacc ACATAATCATGGCGATAAAGGAGCCTG***  ***MaPG3-pro-0800-R: tttatgtttttggcgtcttccat CGTTATCAGCTATGATCTCCTCAGA***  ***MaPE1-pro-0800-F: cactatagggcgaattgggtacc ATTGATCTTTCCTGAATTATGAATCCCTTGA***  ***MaPE1-pro-0800-R: tttatgtttttggcgtcttccat CATGTTCGAGCCGAATTCCTTAGG***  ***MaPL5-pro-0800-F: cactatagggcgaattgggtacc TGTCGTGATCGACGGCCCACATA***  ***MaPL5-pro-0800-R: tttatgtttttggcgtcttccat TTTTGTTCTCCGGTAAGTTGAGAGTTGT***  ***MaPL8-pro-0800-F: cactatagggcgaattgggtacc CTTCTTGAGGATAAGAACACCTACCG***  ***MaPL8-pro-0800-R: tttatgtttttggcgtcttccat CGTCGCCCTCTTCGCCTCTCT***  ***MaEXP1-pro-0800-F: cactatagggcgaattgggtacc GCGGGCTAGTCTTAGGCTGGA***  ***MaEXP1-pro-0800-R: tttatgtttttggcgtcttccat AGTTGAGAGTGAGGGTTGGAGTTGGTTT***  ***MaEXP1-pro-0800-F: cactatagggcgaattgggtacc AGGAGCCTCTTGCAAGCGACAC***  ***MaEXP2-pro-0800-R: tttatgtttttggcgtcttccat CTCTACTTGCTCGAAGAAGACAACT***  ***MaEXPA2-pro-0800-F: cactatagggcgaattgggtacc TCTTTGATACATTCTCACAAGTTGGGAA***  ***MaEXPA2-pro-0800-R: tttatgtttttggcgtcttccat CGGAGCCGTGGGTGAGCAAG***  ***MaEXPA15-pro-0800-F: cactatagggcgaattgggtacc GCTTTCGCCATCCATCCCCCA***  ***MaEXPA15-pro-0800-R: tttatgtttttggcgtcttccat CCTCCTGCACCAAAAGAAATGGTTAT***  ***MaGWD1-pro-0800-F: cactatagggcgaattgggtacc CCGCCACCCCGTTATCACC***  ***MaGWD1-pro-0800-R: tttatgtttttggcgtcttccat GAGTGACTCGCGGGAGAGAGAG***  ***MaLSF2-pro-0800-F: cactatagggcgaattgggtacc CCGTCCGGGTTCCTTGAGG***  ***MaLSF2-pro-0800-R: tttatgtttttggcgtcttccat CCCCGCCTCTTCCTTCTCTC***  ***MaACS1-pro-0800-F: cactatagggcgaattgggtacc GACGATGGCACCGAAAACCAAGAAAG***  ***MaACS1-pro-0800-R: tttatgtttttggcgtcttccat GTGACCCGTTATCTCAGGTACGTACCG***  ***MaACO1-pro-0800-F: cactatagggcgaattgggtacc ATGGAGGTGTGTTTTCCGGCAATGCA***  ***MaACO1-pro-0800-R: tttatgtttttggcgtcttccat GACACGCTCTTTCTTCCCTGATTGCA*** | | ***BamH* Ⅰ**  ***EcoR* Ⅰ**  ***BamH* Ⅰ**  ***EcoR* Ⅰ**  ***BamH* Ⅰ**  ***EcoR* Ⅰ**  ***BamH* Ⅰ**  ***EcoR* Ⅰ**  ***Kpn* Ⅰ**  ***Nco* Ⅰ**  ***Kpn* Ⅰ**  ***Nco* Ⅰ**  ***Kpn* Ⅰ**  ***Nco* Ⅰ**  ***Kpn* Ⅰ**  ***Nco* Ⅰ**  ***Kpn* Ⅰ**  ***Nco* Ⅰ**  ***Kpn* Ⅰ**  ***Nco* Ⅰ**  ***Kpn* Ⅰ**  ***Nco* Ⅰ**  ***Kpn* Ⅰ**  ***Nco* Ⅰ**  ***Kpn* Ⅰ**  ***Nco* Ⅰ**  ***Kpn* Ⅰ**  ***Nco* Ⅰ**  ***Kpn* Ⅰ**  ***Nco* Ⅰ**  ***Kpn* Ⅰ**  ***Nco* Ⅰ**  ***Kpn* Ⅰ**  ***Nco* Ⅰ** |
| **LCI assay** | ***MabHLH28-nLUC-F: cgggggacgagctcggtacc ATGCAGTGCCCAACAGCTCCTC***  ***MabHLH28-nLUC-R:* *acgagatctggtcgac* *CTGACCTCGGCAATTGGTACC***  ***MabHLH28-cLUC-F: acgcgtcccggggcggtacc ATGCAGTGCCCAACAGCTCCTC***  ***MabHLH28-cLUC-R: agctctgcaggtcgac TCACTGACCTCGGCAATTGGTACC***  ***MaWRKY49-cLUC-F: acgcgtcccggggcggtacc ATGGGCGACGATAACTGGGATCTT***  ***MaWRKY49-cLUC-R: agctctgcaggtcgac*** ***TTAGATCGCCATGTCTGCCGCC***  ***MaWRKY49C-cLUC-F: acgcgtcccggggcggtacc ATGCGTCCGGTCTCCCAAGCTC***  ***MaWRKY49C-cLUC-R: agctctgcaggtcgac TTAGATCGCCATGTCTGCCGCC***  ***MaWRKY111-cLUC-F: acgcgtcccggggcggtacc ATGAACGGGAGCTGCAGCGACG***  ***MaWRKY111-cLUC-R: agctctgcaggtcgac TCACCCGGTGGTCCCCACC*** | | ***Kpn* Ⅰ**  ***Sal* I**  ***Kpn* Ⅰ**  ***Sal* I**  ***Kpn* Ⅰ**  ***Sal* I**  ***Kpn* Ⅰ**  ***Sal* I**  ***Kpn* Ⅰ**  ***Sal* I** |
| **Transient Overexpression Analysis** | ***MabHLH28-HA-F: catacgatgttccagattacgct ATGCAGTGCCCAACAGCTCCTC***  ***MabHLH28-HA-R: tattgccaaatgtttgaacgatc TCACTGACCTCGGCAATTGGTACC*** | | ***XcmI***  ***XcmI*** |
| **Transient VIGS Analysis** | ***MabHLH28-pTRV2-F: gcctccatggggatcc ATGCAGTGCCCAACAGCTCCTC***  ***MabHLH28- pTRV2-: cttcgggacatgcccggg GCCACTGCTGCGACTTTCCTGC*** | | ***BamH* Ⅰ**  ***Smal* I** |
| **Tomato genetic transformation** | ***MabHLH28-pBI121-F: gagaacacgggggactctaga ATGCAGTGCCCAACAGCTCCTC***  ***MabHLH28-pBI121-R: atcggtaccctcgagggatcc CTGACCTCGGCAATTGGTACC*** | | ***BamHI***  ***XbaI*** |

**Text S1** Probes in *MaPG3*, *MaPE1*, *MaPL5*, *MaPL8*, *MaEXP1*, *MaEXP2*, *MaEXPA2*, *MaEXPA15*, *MaGWD1*, *MaLSF2*, *MaACO1 and MaACS1* promoters consisting of MabHLH28 or MaWRKY49/111 binding sequence from banana genome. The sequences used for probes in EMSAs were underlined. The MabHLH28-binding sequence is indicated in green box. The MaWRKY49/111-binding sequence is indicated in yellow box. Translation start site (ATG) was shown in red box.

>*MaPG3* (Ma02_g04450) promoter

ACATAATCATGGCGATAAAGGAGCCTGGCAGCAATATGGAGCACCAGCCTGATCCTGCATTCTTAGCAGAAGCTAAAGCTAGCATTAACCAACCAGCAGCTGTGACAACTGATGACAATGCCCAAACTCGGGAGAAAGCAGAATAAGGATTCATGTCCAAGTCAGATATGCTTCTCAAACTTAATTTACATTCGCACTGCAAGTGAAAGTTTGTGTAAGATCATGCAGTAGTATATGACTTCTTTTTCTTCCATTAGGTCAAATTCCTTGAGAAATTTAATGCAAGTGGCGCCCAGATGGAGTGGATTACCTAGAGAAAGACAAACTGTGAACATGATGTTGCCTGTAACATTTTGGTTTGGCATTGCAATAACTTAGGACAGTAACATGGCCATGTGATATTGGTAGTACTTGTGTACCTCAAAACCTTTGTGCAAATTGTAATGACAACATCCATGTTCATGCCTCTGATGTTTTTGACTGACAGCTGTGTTGCTTGACATCTTATTCGATGCTCTCTATTTTTTCATGAAGAATAGTTCATAACAAGTTGCTTTATTCCTCATGATGGGTCTCTCTTTAAGACTTTTTTTGCCTTAGTGGAAGGGTTTGTGTTGTTCAGCTAATGTGCTCTTTTTCCAAGCAGTGATTTATTTCCTATCTTTATCTACCTAATCTCTCATCCTTTCTGCCTAGCCATCCTTACACCACCATATGAACCATTCCTACTGACAATTTATTGGAAATAAATGACATGGTACTGAATCTAGTGAGAGGCACAACATCCCTCCTTAAAGGATGAGGTATCACAAGGAACACTCTTGGCTTATTTTCTGTAATAAATTCTTCTTATTTGATTTCTTCATGGCATGGACAGGTAAGATATGAGGGTCCTTCTCAAGGCCAACATTGAGCTCAGAATGCCATTTTATATTCATTGGGAGGGACAGTTGAAAGAAGGCCACCAAAATTACCAACAACTTCCTCCACCAACCTTGCTTGCATACTCCACCATCTGGTCATTATCATGCTGCTACCACCAGGTGATGTCCATTTCTTTGGCTTCTTATGCATCCACATCACCTCAGAAGTTTGAGGCCAAATGACCTTTGAATCAATGAAATTAGCACTTGATTATTAAAGGATATGACAGGAGGTTGCATTGTTCATAGGGCTTATATAGGAAATGCATCAGTGAGGTAGGATGAGCTTAGACAGGCATCATAGGGGGACCTAAATGCAGTTAGAAAAGGTCAGGTTGGGATTTGGAAAGATGAGCTAACAGAGCTCTTCTACCAACCTTTTCCTCAATAAGAGGAAACATGAGATCTATCTTTTGCCTGATAAAATATGAAAGTTCTTTGTAGGCAAATGATGCATCGTTTTCTGGAAAATATAGCAAGCAGTTCTTTAAGGTGTAAAAAAGCTTTGTTGATATTAAACATCACACAGACCAGCCAAAGTCAAAGATTAAGCAGCAGGGAGGTGTCATGTGTACCAAAACCCATCAATTTGTTCCAATAAAAAAACTTGGATGAATGTGCTATTGGTTTTAGAGGCTCAAAGTCTCATTTGTGGGAGAATAATATTCTGCTACTTTTCAGCTTTTGGAGCCCCAATCCATGTCCAAAAGAACAACAAAAAGAAAAGAGAACAGGAGAGAAAAAGATGCTCCAGAATGCAAAGCTATTGATTTACAATATTCTGTACATTAGAAGGACCCAATTGATCCATCCACTATGGTGCAAAGCCATCAAGTTGAAGCTTGGTATCAGAGACAAAAAGATAAAGTGATGGAGTATACCAGCTGAGCTTTTTCCAGAAGGAAATCCCACAGAACCTGTGAGGTTTCTTGTCTGCTTCTTCACTCTTTGATCAGCACAAGGATCCCAAGTTTAGATGACCAGAAAGCACAAAGATGGGAAGACTGAAAGAAAAGTAAGAAGGGGGGTAGAAAGAAGGGTTGGAATACCACAGCACCAAAGCAACTTTTCTATGCCCACCTTAGACATCTTTAGAACGTATCACATGCGTCCACGCCTGCTCATGTTCTTCTCTTCGCTCAGATGTGCACATCTCCACCCGTCCGCTTCCCATCCAATGCACGGTTTGTAAGACTCCCATGCACTCTTGCTTCTTCCGGAAGAGTTCCTCGTATAAAGAGGTAGAGGGAGAGGCTCCTCACCACCCTTCCTCTTCTCCTCTCCTCTCGTTTCAAGGCACACAGGGGACGAGAAAGAGTGGAGAGAAGGTAGGTCTGAGGAGATCATAGCTGATAACGATG

>*MaPE1* (Ma07_g11280) promoter

ATTGATCTTTCCTGAATTATGAATCCCTTGAAACAGATGATTCAGGAAGAGCGTCCGGTGGCATGCGCGGAGCGAAGACGCGAGTGGAGTCAATAAAAGAAATAATAAATAGGAAATAAAGGAGATACATATTCTTTCATAAACAAAAAGATACTTCTCGTACTCAATCTCGTTTCTTACCCACCACAGATGAGAAAGGCCCCCACAATCTCAAGCAATACGTGTGAGTCGCACTCGGGTAGGTACAATTAGGAACGGCCGTCGTTTAAAACGGGGAAGCACGACATACGTACACGGCCGCCACACGCGTACGAGTTTGCGGGTACGATACTCAAACTCGATGGAGTAGCGGAGTCCCATACACATACACGTTTGAAGCGGTCAAAGCTGCAAATTCTCGTTTCTCTTTCTCTTTTATAAGAATCCAAGGAAAATAATTAAATTGATCGAAAGAAATAAGATAAAAGGCAATTATTTCAGGGAAAAATAATGTAGTCATGCATGACTTATCCAGTAATATTATTATTATTAAATAATTTTAATGAAAATATTAATGGATAAGATTGCATAAGTTGATCTCGAAAATTAGTACTAATCACAATGTAACATTTCAAATAATGTATTGTTTTTTTCATGTAACAAAACCCCACTGGATTGAGAATATAAACATTCCCCGCATCAGCGTTCACATGCATGCGTCGTTTGTCATGAAACCACGTGGATGTATAATAATAATAATAATAATACTTAATGTTCCTTTTACTGCCACTGCACGAGTTATGTCCGCAAGGCGACACACATCTCCATATGCCTAATGTTGTCTATTACGCTGGTAACTCTGCGACGTGAACAGGCGAATGAAAGAAAAAGAATAAAGGGACGCTTTTGCTCTCCTTGATTTAAGCTCATAAAGAGGCTCATTGCATATCCAGTCAGCTACAGTAGACTCCCCCCCATCTTCCATTGTCTCTGCTCCATCCCTAAGGAATTCGGCTCGAACATG

>*MaPL5* (Ma06_g30000) promoter

TGTCGTGATCGACGGCCCACATACTCCCCCGAGAACGACCGACGGGGCCCACGCCGCGAGGGGAATGGTGGGAAGCTCACGTGGGAGAAACATGCGCTGCCGGCCACCTCATCTTCATCATCAACAGAGACGAGTGGAAGCCCACCCGCTGACATGGCACAATCGCAGCGGGCTCGATCGATACAAGTCAGGCCGCGCTCTGCGGCCGAAAGCCATCCCATGTGCTGTGCAGTGCCGTGAACACCCCTATCGTATCCCTTTCTTCCTTGGCATGTGTAGTCACAGTAACAAGATCACGACTCCGAATCCATGGAGCTCCTGCATCTTGGACGAGTTTGGTGTACCCAAAGAGGACGCATTGATGAGCGTGCCGAGATCCCAGGTATTGGTCATGGCAATTACCCCCATTCACCTTTGCTAGCAGAGTAATAACCGTGATTTGGATGCTAAGTCGCTTAGAGACTCTTGGGACTTCATCAATGCTACGGGACCAGATTGAGCCACAGCACCAACCTGCGCTTCTCTCTGGAATAAGGCAGTGGTTGGAATGCGCAAACGCCAGCAGTTCAATCTTTCGCTGCACTGTCTGCGTCCACAATCCATTCTGGGGGTTTTCAACTCTGCCTGACACTCGCCATGTGAGGGCTAAAGTTGAGACACCAGCAGCAATAACTGTTCTCCGTTCCTGGATAACTTAGGGTTCTCATTCATTAATGTTCTGGGCAATTCAATTCGCCTCCATGCCCTCTTTCAGCCCTGAGACACAGTCTTCTCTGATGTTGTACTACCAAAGCTTACTGTAGTATCATGTCTCTGGTTGTTAAGAAGACAATAGAACAGACATGGCAATGGGAGGTAATGTATATATGCATCTGAATAATTATTCTCTCAGTGTTTTGTTCTTGAGGGCATGGTGGTTTGGTAGTCAAATCTACTAAAAGATGAACGGAGAGGAAGAGAGTATGTGTGGAGAAAGGTGCGTCACGTTTCCCATTATCTTTAGCTTCTAAGATTCCCCAAATCACGTACCTTTTTGGACCCCACCGGCATAGATTGTTCACACTACTTCCTCTCTCTTCCTTCTCTCATCCATAGCATCCTCTGCCTTTAAATCCCTTCGTTCCTCTTCGCATTACTCACAACTCTCAACTTACCGGAGAACAAAAATG

>*MaPL8* (Ma07_g04670) promoter

CTTCTTGAGGATAAGAACACCTACCGTCTCAGTTCTCAGCACATAGAGAGAGAGAGAGAGACAGAGAGGGAAGTGCCCAAACTGTGCTGTGATTGCAGGCAGAAGAAGGTCGAGAAAGGGAGAAAAGTCCATGCACCCACACCCGCAGCACATGGCTTGAGGATTTGGCGCACGAGGTCATGTTGCATGCATGTGCAAACCTACTGCTGAGAGAGAGAGAGAGAGAGAGAGAGAGAGAGAGAGAGAGAGAGAGACTTGAAAGCAAGAAGGAAAGCCAACAGTGTGGTTGATGATTTGGGAGTCGGCAGTCACCGGATATGGATGAAAGCTCGAGTTGATCGCTTGGATATTTCATGTTATACTTCACTATTTTCTGACTCTAATTAACGAGTCCTTAATGTTGTGGACTTATAAGTCAAACCTCCCAAGAATCTTTCATATAATTTGGCCCTCATTGTTAGAGCAGAAATTAAACTAAATTATACTTAAATTAAGAAACCCACCAAAAACTATGCCGGAAATGATGACGTCGAGCAACTTTCTCAAACATGACGTTCGAATAGATCACGATCATGATGAGATCGGTTGCGAAGGAGCCGTCAAAGGTGTTAATCCGACGGCCGGAAGACCGAGTTTGCCTGGCGGACACGATGCTGAGTTGAACAGCCGAGTTGCCCTGCCGGATACGTCGTCCACCCCAACGCCGCCATCCATGCGCAGGCTGTGCTCCGAACGCACACGGAAGCGGATGCATGCAACATAAATATGCTACGTCGGCTTGTCGATCCTGAGAACACTGAATTGGCCGTGATTTGTGGCCGTCCGTCATGTGGGCGTGCTCGAAGCATGCCGTGAGTGACTCTGTTCGTGTGTGTGTGTGAAGGAGAACCACATGGGACGTGAATGATGGAAAGAGACGGACGTTGACCAGTGGGCGGCAATGGTCTTCCTCTATGATTAAGTGCACGCCGCTGGTCAAAGAAGAAGAACATTACAAGTAAAGATGCGGCTCATTTGCCGCAAATCTTGGACTTGATCCCAGCTATCCAATTTTTGGGATACTAAGCAGAAGAGAACGAGTGCTGTCGTTTCTCTCTTTTTTTCCACCGTCCAAATTGCATCCGATGGTCACCATTCAAACCGTTCTTTTGATGGACAACCGGAGCTTCCCGGTAGCACGTGTAGGATGGCAATGTCGTCACGGACACGCGTGATGATTGGGGGTTGCTTTGCGGCGACCACGTAATTATGCGCGACACCATCGACGACGACAAACCGTTGTAACGGCAGGCGGGATCGCGTGATGAGGCGAAAGGTGCGGCGGGGAAACCGGCTGCTGCCGGGACGATATCCGACGTGGAGGAGGTGCCTCCCGGGACGAAGCGAAGCCTGGGTCGAGCACGGCGTTAATGTCGGCTGAGGCGACGGAGCACGACGACCCCCTCTCACGTCGTCGGTGGTGGGCGTTGAGTGAGACGCCGTCTCCACTAACGGCGGTTGCAAGGCTGGTAGTTTTCCGTTTCGCGGCTATATAATGGCGCTTCCGCGCGGTGTTTCTCGCCAACTCCTCTTCTGGGAAGCGAGAGAGAGAGGCGAAGAGGGCGACGATG

>*MaEXP1* (Ma05_g07230) promoter

GCGGGCTAGTCTTAGGCTGGAGTTGGTCGACGACGACTAAATCTCTCACGGTGGCGACCACTCGTGGGGACATGCAGAGGAAAAGGTTCGCGAAGACTATCGTGGACAAAGCACTTCTTCCATGTTTTTCTAGCTCACAAGTTTGTTGTACTCTGCTCGATCCGTGTTTGCACTTCGCACCGTCTTCGTGTGCTTGAACTAAGCTACATGAACTAGCAAATATTTCAAAGTGCCGGTGAATGATTGGCGTTCTTAATCTGCCGCGAGAGAGTCAAAGACGGTGGGTAAGTTTGACCCATCCTTTCCGACACTGATACTGCCGAGTACACGTACACTATTGCTTGCGGGATTTGCGGTGTGGCCATTTTCTAGTCGACTCGATGCGTTCGAAGAGCGTCTCTGTCTCTGTCTTCTTCCTCGCCACGTGAAGACGTCGGCAGCCCGGAATAGCATGTGCTCCTCCTCACATGGTGGGACAGTCCGTATTCAAAAAGACATTTTTTTCTGACTGTTTTCTTTCACTCGTAGTGATTCATAAACTATGTTGAAATGGCATTTTAAACTCCGTCGATAGCCATCTTCCCAGCATACACATTAGTTAAGGACCATAATGTTATGGTAAATAACTTTTTTAGTCGCGGCCTCGGGGCCGACACGACTTGTTCGGGGGTCCGAATGGCGGGGATCCTGCGCGGCGTGCCTTGGGTCCTCCTGGCGGTCGACCACGGTGATCTGGATGTCCCGTCAGGGAAGAGCTCTGCCGCAGCGTCGAGGAGGAGGCGACCTCGTCTCGCACCTGCACACTGGTCGGGTCGGAAGCTCAGCTCGACCCCTCCGACGATCAAGTTAGTGACGTGGAGAGGATGATGAGAGAGGGAATGCTCCTCTGTGTCCCTTCCCCCTTTTGTTTAGAACTCGGGGTATTTGTAGTTGGGTTTGATGTTACCTGATGTGCCCGCTTGCAAGGGCAGGGTCGTACCTCTGATGGCGTCTGACATTACCGTTGGAGTAGCGTATAGGACCGAACTGCCGCAGGGTATGGGCGAGCCTCGGTTGACGTCTTTCTTTGCTTCGGCCGAGCGCGCGGGGTCAGACGATGGAGTTGTTGGCCGAGAGTGGGTGACGTCGGCGCATGTCAAATCGGCGTTAATGCCCGCTTCCTAGGGCAGTGTGTCGTCCAAGTGTGACGGATGTCGTGGCGTATTACGTCAGATTTGTTTTTACCCCTATCACATAAGGTCTTGCAACACACACTATTGGTTTAATCTAATCGAACAGAGGAGGTGGATAGCAGCAACTCATTGTCTAATTATTGCTCATCTTCCATTGTGTAGTGTAGTTTATTAGCGTACTGACATGATTGCGGCAATTAGTTTGAGCGAGTCATAGTAGCTGGAGCGTAAAGAATGGAGTAATCTCGGCGTGATTGATTGAAGCACGCATATTTGTGCCGCAGTATGGCACCGTCGGCCATACAAGTCCGATTGAGACCCTTTATTTCTTGAACTGCTTCGGCGCGCATCTTTGGATTTCCAGCTCCGATACCTATGAACACAGCCTTCAAAGAGTGAGTCATCAATGTGAACTGCGCAACTTCTTCCTCTGCACGCCATTCACATCATCTTCCTCTGCTTCTGTTCCATCAGCGAGACTTATTTGCGCTTCCAACTAATCTTGTCTTGTCCTACCCTCTCCCCATCTCCTTTCCTATTGTCGAATGTAAAGAGAACCCCAAGAGAACACAACAGGATAAATGTGGCACCATGAAGTTTCTTCCATGAAAGCAGATGCATTTGGGTGAACGAGAGGACGCATTCGGACTGTAGTTGGTACTGTCACCGGGACGATTGCATGTGCTCCTGATTTGGATGATCGCTGCGGTCGTCTTCAAGCCTATGGCTGGCCCATGGCCGCCACCAACTTGTCTCTTTCCAGAGATTGAAGATG

>*MaEXP2* (Ma11_g21280) promoter

AGGAGCCTCTTGCAAGCGACACGTGTCGTGTCCATGCACGAATCTAGAAGCATTTACCCTCCTAAATGTGAATGAATGTATGTTAAGAGTAAGAACTCGACTGACTCGACCACATCGAGAATGATGCTGTCCGGATTTATCTTGCGTCTTATACTCCACAAAGACTCATTTGGCTCATCGTTACAATAAATAGTAATACCTTTCTTTTCCTTTTGATTAATATAACAATCATTGGACCGATGAGTATGAATGGCAGCACACTATGCAGGCCTTGGGGAAAAGTAACCCATGTAAGTGTGAATAGCATGCATGAGTTAGGTTGACTTCCACTCCGCGTCGCACATGCACCTACACACTACGTGAAACCTAACTTGGTTGTGCCATTACGTGACGAAATAGTCAACTCCTCTCTCTCTCTCTCTCCTCCCCACATGGGTTGGTTCATAAGGTGCTCCGGCGGCATGTGCTGCAGACACGAAGTCTCACCTCTCACGCCACGCACGAGTCACAAGTCATCTCCCTTTCTGTATCCAACAACCACCGTCATACTATTCTCGCATATGGACATGCGAATACGTAGAACATGTGGTTGCGATCTCGTCATCTACCATCCGTCTGCGTCCATGATGTACGGGAGCCATGTGCTTGCAAACCCTCAGAAACTAACGACGAAAATAATACTTAGACAGAGCACAACACTTGGTTGACTTGATTAAGTTACTTATGGTTCCGTTTCTCTATGCTTCATAAGGTAGAATGTACGGTTACGTACACCGTTTCCGTCTTCGGGTGGAGGCGGCGCAAAAGGTGGCCAAGTGTCGCCGGCACCAGGCTGTAGATTTTTGTACGAAGCTTATTTGTATATAGCTTTGATTAAGCTTCACCAACTTGTAATAATTTGTTCCAGCGCGCAGGTCTTATAGTTGCGTGAAGTCAACGTAGACTAAACGCCCAACACACCGTCAGGGTCGAAGAAGAAGAGCTCAGATTCGGCACTATATATAGGAATGAGGGAGGTGGGATGGTATGCATTAATATTCACAGCACCGAGTAGTTGTCTTCTTCGAGCAAGTAGAGATG

>*MaEXPA2* (Ma05_g07240) promoter

TCTTTGATACATTCTCACAAGTTGGGAATTGGATTATACTACCAGACCACAAGGTAATGAAAATATTTCTATCTTGTTATGCCTCCATTAGAGACCATGTTGGGACAAAGTCTTAGCAACCACATGGTGTACAAAGTCATCGATCATGATATGTATGTATGATTAGCTAAGGATGATAAACTATTGCAACAAATACTAATAGTTTAATCTGAAGGAATGGAGGATGTACCACTGACTCATCATACAATGATCGCTCACCCACCTTTAGGTAGTGTTATATCTATATGTGTGTGTGTTGAGGTGATTGTGGCAATTTGGAAGTGCTGACACACGTGTTCATTATTAGACTCATTGTTTTCATATAACTGGAGCGTGATGAGTGGAGGTATTTTGGCGTGATTATTGAAGCACCGATTCGGATTCACACGAATCTCAAAGATGGTTCCTTGTCCTTCTACTGAGCACTTGTGCTGTGATGCACCGTAAGCCACATAAGTCCAATTGGATTCCTCCCTTCACAATGAACATGAAGGAAGGGTTACAAGGCTTAATTATCAAACCTATGAGAATAGCCTTAAAAAGTCAATGTGAGCTTTAGAACCAACGTAAGGGATGACGAGAGACAGTGATGCACGCCATCGCTTCTTCTTCTTCTTCTTCTTCTGGACTTGTTTGCTCTTCCAACCAATCCTACCCTTTTATAATCTCCATTCCTTTTGTTGCATGTCATGAGAACAACGCTACGTTGGTTTTCACCAGCAGTTCTGATGCATGTCCATCGATGATAGAATCCCAAGGGAACATTAACACGATAAGTGTGGCAGACACCATCTAAATCTGTAGAGGAATATTGGTTTCGGTTCAGTTGTTACCGTGAAGTAGATTGAGACATGCAAAAGGAAGGAGGAGGAGAGAGATGAAGCAGATGCATGTGGGTGAGAGGGAGGGCCCAAAGGCTTTGTAGTTGGTACTTCGACCGCAACAATTGCATGTGCTCCATGATTTGGACGATCGCTGCAGTCGTCTGCATGTCAATGGCTGGCCCAAGGACCAGCAACTACCTTGTCTCTTTCCGGAGAAGGAGAAGAAGAAACCGACTCCAACACCCCGCGCTCGACCAGTATAAAGACACCGCCCTTCCCCTCCCCTTCGCTTCACAACCCAACCAACCACTTCTCCCCTTTCTGCTTGCTCACCCACGGCTCCGATG

>*MaEXPA15* (Ma06_g12190) promoter

GCTTTCGCCATCCATCCCCCACCCGCAGCTTGTCCGAAGCTTTTGTTACCGCGGCATGTGGCTTCCCATGTGCGGTGGGTCGACGCATCCGTTGCCTTATCGAAACATCCAAGCAAGAGCGGCTTTCCGTCGAGCAGTGGTCGAGCATCAGACCTCGAAATTTGGCAGCACACCAAATCCATCGTTTCTCTTCTCTTCACTTCCTCGACGGAAGTCAAAAGGGAAGTAACGTGCGTCACATGATACCGACGACTTTTGGGGCCTGATGGGAGCCTACAACTCACTGGCAGTGTCCCCACCAACATGACAAGTCGTGGATGTTCTACTGTTCTCGTGTTCTTCATCCATGTCTCCTTCCTCGAGCGCGTGACGTCCATCTCATTAGCTGGACACACAGCGTGTGGTAATGAGGTATGCCTGCCTTATTACCATCCTTCGCAGACGATACACATTTGTAATCAACGACGATCTCGAAAACGACTGTGCACGAAGCTGTCCTCGATCGGTCGTCTTCGTGCTCCGAGCCCTCCTCGCGACCAAGTTATGATAAGAACATGACAAAGTAGACCTACCTGATCCATGAGGTAACGATCAATGATCCGATCCATCCGAGTAAAGTTCTTTACGTCACTTGAACAGTCGTCGTCTCCTCAGACACAGGGAAAGCCAAATCACATCACCTGAGCTCATTCTCACTATTCTAATCCATCACGACAATCTTGGAAGATTCGCAGCATTCTTTTCTTGTTGGCTCAATCATACGACACTAAGGAAACATTTTGAATTAACCATCTGTCCGTAGTTCGATCTTTTCAGGTTAGAGGACTTCTTGGATCATCGACTGCTGTAAACTCATTGACCTACAACTTTTTCTTGACTCAACTGCAGGCAGTCAAAACGAAGAACTTTGGTCAGTAAGAATCGACTTCATCCCTCCCTTTTGCTCATCCCATCTCACCGAATCGAGTGGTTGGTCCTGACGTCGAAAAGTGGTGCTAAGAAGGCCCACCATATCTGTGCAAAGATTCATCTCTCTCACTGACAAAAATCTCTACTGTTGCTTCAAGACTACACACGAGCTCAACGGTAAGTGTATCGCATCCTCATCAAAGAGTAAGCATGAGCATAGCAAAGTGCTCCTCGAGAATGTTTCCGGAGATGAGAATGGTTCCGCAAGCACAAGATCTCTGTGATCTATGTTAAGCATGCTTGCACCGACAGCGAAGTCCCATACCACCTAACTATGCAGGACAGAGATCCATCTCGACTGGTTCAAGATTTTGAGATGCAAGAACATAATCCTGCTGTCGATAGAATTCCTCTGGTTGAACTTTTTCTCTCAGACTCAGAAACAGGGCAGACAGTATGGACCCGAAGATCTAATCAGCAAATCAAACTGATATTATAAAGTAAGATCAAATGATTATCCTTGTAACACACCTTGTGGGAAAGTTAAAGAAAGTGAAAGGAATGGAAGGTGGTGGTGGTGGTTGGAGAAGGCATCATGTAAAGGTTCCAAAGGTGAGAATGTCGGTTTTCGACAGTTAAATCTGATCTGAATTATTTGGCCTTGATATGAGGTATGGATGTCATGGAGTGAGGTATTATGGGAGCTGCACACGCAATTGAATGGAGTTAGACAGCAGTTGGCGTCTTCTTTCATCAGATGGAATACTGAAGCTCGCAGCGAAGGAGAAAGCTATAGGAAGAAAGGAATCACAACTGGCTTTATCTGTCTTTATTGCCACACTAAATTTAAGGACATTATCTACCAAATGAAAGAGAAATGAAGGGTCCATCGCGCGTGGATCTTGACAACTTTGCCTTCTCTAACCCTAGCTAGAAGACACGATCACATTCCGTGGGTGCTGGATCAAAGGGTGGTCAAGTTGATCTCTCACGGATGACCTCAATCTGTACTGAGACCAAAAATGCCGGTGTGCACTCCAGCTCTTGCAGGCAGCTGTGCTGCTGGTCCCCCGGCGGTGCGAGGTTGTGGCCAGTATCATAAAGTTAGGGTAAATCCAAGGGGGAACCTACCACTTCAAACCTCTATTTATGCCCCCTCGACTCCCTCCCTCTCCCCTCAAGCCAATCAAGCCTTCCTCTTCTCCTCTTGTTCTTCCCCTGGCCAACACTCATTGTCCCAATGTAGTCATCTTTTGCTCATCGGTAACCTCACACGCATGGTGGTCTAGTGGATAACCATTTCTTTTGGTGCAGGAGGATG

>*MaGWD1* (Ma03_g15660) promoter

ATTGATTTATTATGAATATACCATGTTAATATCAATTTAAATATTTTGAATAATAGTTTCGACTTAATAAAGTTAACAAGTCAATGACCCCATTAGATCGGATCATAATAATTAATATTATAATTGATTAAATATTGAGGCATGATGAAAAATTGAAGTAAGAAAAATTTATTCGTACAAAAGACATTTCACATCACTAGAGAGATCGTAAGTTATGCCTTACATGCATCACATAGTAGAGTCATCACTGTCTTACATGCATAATATTTTGAATTTGATAGATGTTATATTAAATCTTAACAAAAATATCAAATATTTAAGTAGAAGAGATTATAATATCTCAATTAGTCTCACATTCAAAATAAAATTATTAACTTAAGTTTAAATATTTTTAACCAAGAATCACTTATCTCATTAGATGAGATTGTGATACAGTGACACTAATTTTCCAAACTTCATTCTTCTCGTTAGATATATCATCCCATGACTACTTTAATACCAAGTTAAATATGATTCTAAATTTCTTTTTTACGTAATCCGATTTATTTTCCAACGAGAACATCATGTTCGTTCAAAGAGTCCAAGTTTCCTGCGGAAACCCAAACAAATAGTAGGCTAAAGAAAAGAACACAAAGTAAGCGAGTGAGATAAGCGAGCGCCCGACATAATTATCTATCGTCTTTTCTTGCGGAATGGCGGGCGAGCGGGAATCGCTTCCGTGGTAAGCAAGTAAGTGTCCCTTCTTAGCTTAGCCCACAGTGGCGTCCACCGCCACCCCGTTATCACCTTTGTTTCTTTCTTTGGGTTGTGGGTCCGCCGCCCATGGGAATTTGCCCAAAGACTGGACCTTCCAACGACACCCCATCTCTCTATGCATTTAGAGAAGCTAAGTGCGAGGAATAACGAGCCTTGTTCTTGGAACAAGCGCCATTGCAGATGGGGTGAGGAGCGATGGTTTACATGCTAACTCAGATGTATCTCGGATTCGAATTTGTCGGATCAATTATTTTAATTAACCTGAATTTTCTTTTTTCCTGTCATAATTATCAAGTGGTCTGTATAAGGCACTCATTCGATATCGATAGATGTTCTTCTACCCTTCCCCCCACTTGCACAAATATCACGGATAGTCACAACCTTCCCGTGCCGTGCCTCCCACCCCTCGTATAACAATCTAATCACATGATGGTGTGTGGCCGCCATCATTCTCTCCCAAGCAACCACGTGTAAACAAAAATATCACGCTAAGACTCCTCTATTGTCGATCATCCTTACGGTGGGGCTCTCCTTAACTTACTCGTGTCCAGAACCAGCACCTCATCCTCACACACCAACACACCATTCTCTTCCACTACCAGCACACCACGTGGTAAATCCGGTTCACTCCGCCCTGGCATCCTGAGGTCGTCCAGGAACCAGGGATGTCCCTCCAACGAAAGTATGCTACGTGAGCCGTACCACCCGGTTCTAGTCACGTGCGTGATCGGGGCGTTGATCTTTTCCCCGCCTCTCTCTCTCTCTCTCTCTCTCTCTATCTCGCTCTCTTTCTCTCCCTCCTGCGAGTCACTCTGCGCCTCTCTCTCTCTCCCGCGAGTCACTCTGCGCCTCTCTCTCCTCCGTCGTCGTCGTCCTTCTTTTTCGGTTTCTTCCGCCAATCACCGCATCGCTTTCTTCTTTTCTCCTATTAATACCCTCCGAAGTAGAGCAAGCAAGAAGCTCTTCCTTCAGGAGGAACGTCTCTCCCTCTCTCCACTTCTCACACCCCCGCCCCCGACTCAGCGAATCGACATTCTTGGCGTGTCAAGGAAACCTTTCTTTTATTTGCGAAGGTAAATTTCTTAAGTGAATTCTTCGGGGTTCGAAAACCATAATCTCCTCCTCGTTTATTTATACCAGGAACAAAAATTCAGAGATCAAAAGGAAAAAATAATTAAGTCTCAAGAAAAATTGAGCTTATTTGTTTATGGCCTTCTTCGCAATAATTCTAGGGTTCGCCCTCGAGCATTATATAAATCTTAGTCACACTTCCATGACCATTCGTCCTCCTGCTTGATGCGGTCCCTGGAGCTGCAAAGTCCGATTTTAACGTTTCCTGCTCGTTCCGGTTCTTGGTTCCGCTGGACATGAAATAAAGCCGGGCCAGTAGTCTAGACAAAATTTTGATTAATCTTACTTTCTTCATGTTCTTTTTGTCGGAACTGGTGGGTTCAATTGTCAATCTCAACTTCTTGATATTTTTGAATTTTGCAATGCCAAGAAGATCCATGTTCGATCTGATCGGATGTCCTTAAGATTAATTAGCTTCCGTTGAGTTCGTTGATTATGACATTCTATCAAGTCCATGTACTCAAAGTTCAAATATGTGATTTCTAATTATGCTTAGGTCCCATGTCCCAGAATTCCTTACGGCCACAAACTGTTTCAGTAAAGTTTTTGTTTGCGCTTTATGAATATTTTCATATATCAGAATGGTCATGAATTTTTCGTGAGGACCAGAAATACAACTTTCTGATTGAATTATTGGTTTTGATGAGATCCTTCATAGCTTTCTCCCATGCAGGGTACCCTGCATAGACTTTGCATTCACCTGTCCTTTTTATTTCCTACTATATTATTTCTAGAACTAGCACCAAGGTAACTTCTAGTTCGTGTAAAGCGCTACATTCAATTTGAGCTTCAGTCTGCATTTAATTACCTTCTGAAGTTGTTATTGCTGGGTTTAAGAGTTGGTATATGATACAGGAACATG

>*MaLSF2* (Ma04_g00190) promoter

CCGTCCGGGTTCCTTGAGGAGGATGAGAAAGAGGTTGCCAGCTCGACGGCCGCCTCCCAACACGAGTTCTTCTTTGTTTGACTTGGAACGAAACACCCATGGGAGGGTGAGTGCGGCCCACACAAAAGGAGTGGTTGGATATGGTGGGGCGTTAGGAGGACATGAGTCCGCAGGGGCCGAGTCGAATCTTGGCGGTGTCCAACCACAGCACTAAAAGAAAAGAAATGGATGTTCTTTCTAATGGGGGTTGAAGGAATGACGAGGGTTCCACCAAGAGACATCATGTGGCCAAAGGTGGGTCAATGGACTTTTTGTTTTAAGATGCAACTCGACATGCTTTGCTTTGCTTTGTAAACGAGCTGTGTTCTTCTCGACTCGACGACCCACGCAACACTCGATTTAAGAGTCTGAATCACTATTTTTTTTTTCTTTTCTAAAAAATAAAATAAAAATTAAATTTATAATAAATATATTTATTGAGTATAAATTATGTTGGATGAGGTTTCATAATTAAGTTTGAAAAGAGTTATTTATACAAGCTGGATGGAGCCTTTTTCCTTGGTCAATTATACGAATGAATGAATGGCTTGCTGATTCAAGTTGGATTGCATTTTGCATCAGAACAATATCTTTAAAGATAAAAAAGACTTCGACACTAGAAATATTAAAAAAGATTCAATGTAATGGCTCAAATCAATGGGCCTGTAGAATGGGCTCAAATCAATGGCTCAAATGAATGGGCCTGTAGGCAGCCCAACAAGCCATGTAATGGCGCCAAATGTTGTCCTGTCACGACGCCACCGTCAAGTTTCCCGCCAGAAAGGTTGCAAATATCACGTGCGGGTTAAGCCCGAAATATTAGAACTCGACGACATGTGCTCCAGAAAATTCTACCAAAAATATCTCTCTCATAATTATATTTCATATTATACTTATCGTTTGCACAACAACAAAAATAACAAATAAGATCGTAATTTTCCAGCTATTTAAATCTATATTCTTAACTAAAATAAAAATATTTAAATATTTATGATATGAAAATTTATATGCCCGACCCTTACTAATTGTTTCATATATATATATATTATGTGTGGTGGTTAAAATCAAATATAGAGATAAATTATGAGGGTGAATATACTATTAATAGTAGAATTATTTATCTTTGTTCAACTAAAAGGGTCTAATTGGTTACATTACATGGCTATAAAGTGACTTTACAACGTGGTCTCTTCCGAGGCGGAGGCGGCGGCGGAGGCAAAGGCGAAAGGGTCTGTGACGAGAGCGCGGCGATCGAGAGAGACAGAAAGAGAGAGAGAGAGAGAGAGAGGAGAGAAGGAAGAGGCGGGGATG

>*MaACO1* (Ma07_g19730) promoter

TAGCTCTGTTTCTTGTGCTCTTCTTTCACGTAGATTCATAGAGTAGCTTAAGTTGTTATAGATTACCTGCTTGCATCTTCTTCCTGTTTTCCTCTGTCAAACTGTTCTGTTCATGATGAGGCAGCACCGAATCTAAGAGAAATATCCTAATGTTGATTGATTTAACCTCATAAAACTTGAAGCAGAATATGCTTGCCGCTTTCATGTGATCAATTGAATTGTTTGCTTGCTTCACGAGAACACCACATTCTGAACCCATTGCTTTCTTGTGGCCACCAACCGGAGAAAGGGAGTCTATATAACTAGCCGAGCGAGGATTTTCCCATGACCTGTTCATCTCACGTAGAGATGGTGATTTGGTTATAGTTATAGCGATCTATGATCGAAGAATGAGAAAATACCCAGATAACGGAGATCCATGCGTCACCAGATGGAACCTCGGCCGAGTGACACTGTTTGCACACCGGATACTTCATGTTCACGGCAATGGCCGACATGCCGAACAGCCATCGAGCGTTGAATGTGAGGCAGGAATGGCCCATTTCTCACATATGAGAGGGATACGAGTGGAAAGGGCGCTCTAATGAGCTGTGAATCGAAACAATTTCTACCTATCGATCCCTGTTCTTTTGATATGAAGTATAGCCAACAGGTCAAGAGAAGACGAGTACACACGCATCGCCGATGCTGTGAGGTTACTTTCTGAGGTTGGCAATTTGTCACTACAATCCAAGCGGAAGCCATGCACGCGAGGCGTCGCCATGGAAGAACTCAACAACATGATGCCTTCCCGGGTCTCCTCAAAGGGGAGAGACCGATGGAAGCAGCCAAACTTGGTCCCCGATCGTGATGGGACGCGAGAGGTGGAGGCAAGGAGGGTGGAGAACCAGGCCAAAGGTGGTGGGGCTGAGAGATGGCCAACTGGGTCACCTTATGGAATCGGCTCCGATACGTCTTCCACTGCTGTTGCTCTCGTCGATAGATCCTTCTCCAACTTTGCTTCTTCATTCATTTCGTCCCTCGACGTCAGGAACGCCTATAAATTGCCTGGTAATCAGCAGCACCTAGCACACTCCAGATAGAAAGCACAAGTGCAATCAGGGAAGAAAGAGCGTGTCATG

>*MaACS1 (Ma04_g35640)* promoter

GACGATGGCACCGAAAACCAAGAAAGAAATGGGGATGTCCAATCTAAAACGTTGCCGACATCTTAATCGTGCCTGAGATCATATATTACCTACAAACACACGTTATTTATTCTGGCTTTCAGTGGAATAATTACTCTTACTTTCTTTGATGGATTGCTGGAAATGGACTGTTTGGATTGGCGAACGAGATTATAAGAACAACATTCTACTTCAAATATTAATTTCACTTTTGATACTCGGAAACTACACCCCATAAATTTGATCTTTGAAGCCTAAGAATTAAATCTAAATAATGGTAGAAGCATAATTTATTTCCGGTAGGTGGATGGCTTACATGCTCACCTAATAATTTATTTAAGTGGGATCCATGAATAGGCCACGTTGCATTCGTACTTTGATGATTACTGTACCATGAGCTGTGTAATGCGTGTCTTGATTGCGACCTCCCGTAGACCCATCAATATTAGATTGAGACGAAAATAACGTCGGTGAGTAGACAACATAGTACAGTAGATCATGATAGAACAATTCGAAATGATAAGACATACAAAAAAATTTGGTATACGACTCGATATATAAAAACTAAAGATATTTCTTTCATCTATATATATATATACAATTTGTAAGCTTCCTAAAAGAGATTAGATTTTGGGTCAACTATAATTGCCTTCCGAGAAAGTAGAACGCCTCTATCAGAATCTCACGCCTACAAACAACACCAATCCTAATCATTCACATGATCTTAGTGTTAATGAAGTAGCAAATTCCAAATCACATTTAAATAGCCGGTCATTTCAAACATGAAGATCACGCTTTTATTGTTTCTTTTTTGGTAGAAGAGTTAATGAGATCCACATTTTCCTTAAGAGGATGCATATAAGCCAATTAAATTAACACCCATATGTATAAAATCCAATTATGTAAAGGCAAATCGAAGTAGTAAGATACGTTTCTATCTGATCCGATTAATATTCTCTTTTAAGATTGATAAAGAATATATGCTAATCGATATGGGAGGCAATTCCTGACGTATCATACTCATCCGATCGATACGTAATACCGCTAGGTTGGTGATGGAGATGTTAATTCGTCTCACGAGATCGAAATATATAATGTTGACGAGAAAAATTGACATGACAAGTCAGTTATTCTATAACCATAGCTTTGATTTTATGACTCATCGTGCGTAACTCTTCTTGACATTTATTATCGTCTTAATTTAATTATCATATATTATAATATAAAAGGTGTTCTAGTGGGAGTTTTGACTGAATACATTATTTAATTTTAATGCTTGAATGAATTCTTTAAACTTTTACTAACCTAAATATTGATTGAATAAGTATGCTTCGACACGATTTTTCTACGTGATCATTGTTTAAATCTTTGACCTCTTAAACACCTCAGTTTGAGTCAAACTCGGAAACTCGAAAGTGGACCTCCGTCAGACTCCTTTAACATATGTTAAACAACTTGAATCTAATTTGACACTAATCAAAATCAGACTTGATTGAGCCTAAATGGATCTAAAAATTAAGAAAAACTTTGTTGAATTTTCCTTCTTCGCAAATAGCATGTCATCGATGAGATTAAGGTTTACAAAGAGCGGCGCACAATTTTGTTTTGGGATAAATAATTCTGTGCTTACAATATAGAAGAGTTCGAGTCGAAAGCGACTCCCGAGTTCGGAACACGTCATTGTTGCTAGCAACACTGAAGCTTCCTATTTGGCGTCACCTGTCGATGTTACGGCGCATCCATCGCCAATCACGTCCATGCTTTACACGCTGCCGGATCGACTCGGTTTTCATGTCCTTCTTTTCCAGTCTGAAGTCCTCTGTGTTGACCTCTTTGGATGTTTGAATGGTCTCGGGATTTGCCTATTAATGGTCATCGGAATCGACTCTTGCAAACTGCAGCAGCTGCTTCTCCTTCTTCTCTGCTCGCTTCAGCCTTTTCCGGTACGTACCTGAGATAACGGGTCACATG
